# Magnetically Guided Microcatheter for Targeted Injection of Magnetic Particle Swarms

**DOI:** 10.1002/advs.202404061

**Published:** 2024-08-09

**Authors:** Harun Torlakcik, Semih Sevim, Pedro Alves, Michael Mattmann, Joaquim Llacer‐Wintle, Maria Pinto, Rosa Moreira, Andreas D. Flouris, Fabian C. Landers, Xiang‐Zhong Chen, Josep Puigmartí‐Luis, Quentin Boehler, Tiago Sotto Mayor, Minsoo Kim, Bradley J. Nelson, Salvador Pané

**Affiliations:** ^1^ Multi‐Scale Robotics Lab Institute of Robotics and Intelligent Systems ETH Zurich Tannenstrasse 3 Zurich 8092 Switzerland; ^2^ Transport Phenomena Research Centre (CEFT) Engineering Faculty Porto University Porto 4200 Portugal; ^3^ Associate Laboratory in Chemical Engineering (ALICE) Engineering Faculty Porto University Porto 4200 Portugal; ^4^ Experian LDA Porto 4200 Portugal; ^5^ FAME Laboratory Department of Exercise Science University of Thessaly Trikala, Karies 42100 Greece; ^6^ Institute of Optoelectronics State Key Laboratory of Photovoltaic Science and Technology Shanghai Frontiers Science Research Base of Intelligent Optoelectronics and Perception Fudan University Shanghai 200433 P. R. China; ^7^ Yiwu Research Institute of Fudan University Yiwu 322000 P. R. China; ^8^ Departament de Ciència dels Materials i Química Física Institut de Química Teòrica i Computacional University of Barcelona Martí i Franquès, 1 Barcelona 08028 Spain; ^9^ Institució Catalana de Recerca i Estudis Avançats (ICREA) Pg. Lluís Companys 23 Barcelona 08010 Spain

**Keywords:** magnetic catheter, magnetic control, magnetic nanoparticle swarm, magnetic nanoparticles, targeted delivery

## Abstract

The initial delivery of small‐scale magnetic devices such as microrobots is a key, but often overlooked, aspect for their use in clinical applications. The deployment of these devices within the dynamic environment of the human body presents significant challenges due to their dispersion caused by circulatory flows. Here, a method is introduced to effectively deliver a swarm of magnetic nanoparticles in fluidic flows. This approach integrates a magnetically navigated robotic microcatheter equipped with a reservoir for storing the magnetic nanoparticles. The microfluidic flow within the reservoir facilitates the injection of magnetic nanoparticles into the fluid stream, and a magnetic field gradient guides the swarm through the oscillatory flow to a target site. The microcatheter and reservoir are engineered to enable magnetic steering and injection of the magnetic nanoparticles. To demonstrate this approach, experiments are conducted utilizing a spinal cord phantom simulating intrathecal catheter delivery for applications in the central nervous system. These results demonstrate that the proposed microcatheter successfully concentrates nanoparticles near the desired location through the precise manipulation of magnetic field gradients, offering a promising solution for the controlled deployment of untethered magnetic micro‐/nanodevices within the complex physiological circulatory systems of the human body.

## Graphical abstract

This research showcases the targeted delivery of magnetic particle swarms using a magnetic microcatheter with a particle reservoir for precise ejection. Once ejected, these particles are swarm‐controlled by external magnetic fields, even in pulsating flows. This system shows significant promise for intrathecal magnetic therapeutic agent delivery within the central nervous system, as demonstrated with a spinal cord phantom model.

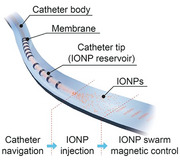

## 1 Introduction

Precise delivery of therapeutics can optimize treatment efficacy while reducing side effects.^[^ ^1^ ^]^ Small‐scale, motile magnetic devices have emerged as promising biomedical platforms as they can be used for targeted drug delivery,^[^ ^2^ ^]^ imaging,^[^ ^3^ ^]^ and localized diagnosis^[^ ^4^ ^]^ in difficult‐to‐reach areas of the human body. These magnetic drug delivery devices range from magnetic nanoparticles^[^ ^5^ ^]^ and nanoswimmers^[^ ^6^, ^7^ ^]^ to magnetically guided drug‐loaded liposomes^[^ ^8^, ^9^ ^]^ and therapeutic cargo microrobots.^[^ ^10^, ^11^ ^]^ Magnetic particle swarms have emerged as promising microrobotic delivery platforms^[^ ^12^, ^13^, ^14^ ^]^ due to their reconfigurability and adaptability, enabling them to navigate through challenging obstacles, such as constrained vasculature conduits, by adjusting shape in response to magnetic input. However, a major drawback is their tendency to disassemble due to fluid flow forces, caused by their reliance on magnetic dipole interactions.^[^ ^15^ ^]^ Additionally, the small size of the particles makes them prone to opsonization by the body's immune system.^[^ ^16^, ^17^ ^]^ Consequently, finding the optimal implantation procedure is important to maximize the effectiveness of these swarms and to avoid compromising the delivery system's precision and safety.

**Figure 1:**
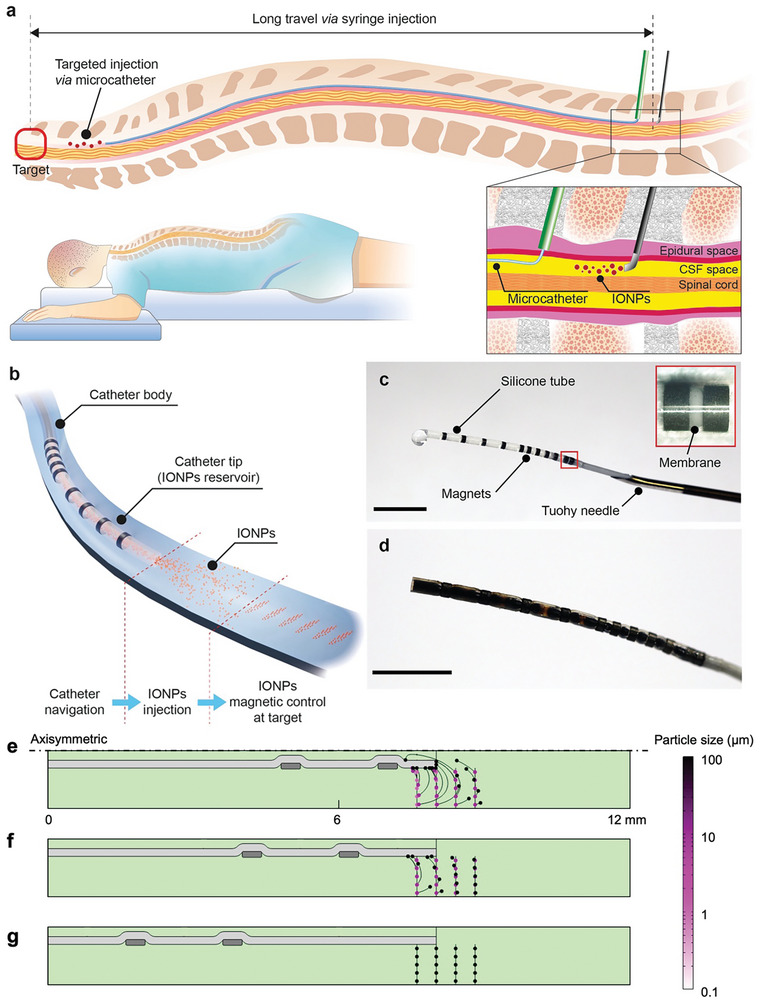
a) Schematic diagram comparing the performance of the catheter injection method and syringe injection method for small‐scale magnetic devices delivery in the cerebrospinal fluid (CSF) space. b) Schematic diagram of targeted iron oxide nanoparticle (IONP) delivery strategy using the magnetically guided microcatheter. c) Image of the microcatheter. The microcatheter is inserted through a Tuohy needle. (Scale bar: 5 mm). d) A reservoir is loaded with IONPs (Scale bar: 5 mm). e–g) Numerical simulations showing the magnetic attraction of the particle aggregates close to the catheter tip, in a scenario without ejection and counter flows. The distance between the catheter tip and the ring magnet ranges from e) 1 mm, f) 2 mm, to g) 5 mm. The distance between magnets is 2.5 mm.

When delivering therapeutic agents to the central nervous system, a common procedure involves intrathecal administration. This method entails injecting drugs into the subarachnoid space, where the cerebrospinal fluid (CSF) is located, typically through a lumbar puncture for the safety reason (syringe injection method in **Figure** **1**a).^[^ ^18^ ^]^ If the target site is, for example, 0.5 meters away from the puncture site and the mobile magnetic devices move at a speed of 100 µm s^−1^, it would take ≈1.5 h to reach the delivery site. This timeframe is inadequate for practical applications.^[^ ^1^ ^]^ Additionally, this method has difficulty in efficiently targeting therapeutic agents’ carriers to specific areas.

Therefore, optimizing intrathecal administration using magnetic micro‐/nanodevices is key to shortening delivery times and controlling swarm behavior under pulsatile flows, such as is observed in natural CSF dynamics. Recently, our group has successfully demonstrated the use of artificial microtubules for the assisted delivery and collective transport of magnetic microcargoes.^[^ ^19^ ^]^ Similarly, microcatheters, which have been already utilized as a retrieval device for collecting small‐scale magnetic carriers,^[^ ^20^ ^]^ could also serve as valuable tools for intermediate delivery assistance. Specifically, magnetically guided microcatheters, as discussed in,^[^ ^21^ ^]^ are tethered devices that enable minimally invasive procedures during medical operations, as they exhibit tailored steerability under magnetic control, and additional functionalities, including the ability to adapt stiffness on command.^[^ ^22^, ^23^ ^]^

Here, we propose a magnetic microcatheter for delivering magnetic nanoparticle swarms in close proximity to target tissues (catheter injection method in Figure 1a). The magnetic catheter is equipped with a reservoir of magnetic nanoparticles, which was engineered to efficiently expel particles against both the magnetic attractive forces generated by permanent magnets positioned on the catheter and the drag forces induced by physiological flow conditions. Optimization of the ejection flow was achieved through numerical simulations integrating microfluidics and magnetic fields. The magnetic steerability of the catheter was assessed using the electromagnetic navigation system (eMNS). Subsequently, we demonstrated the magnetic navigation of the microcatheter, the ejection of magnetic nanoparticles, and the collective motion control of the nanoparticle swarm within a spinal cord phantom simulating pulsatile flow conditions to mimic physiological CSF flow dynamics. The main goal of the presented strategy is to enhance the efficacy of delivery by both reducing the delivery time and boosting the precision of targeting to the intended area. While the former was accomplished via utilizing a customized magnetic catheter – that offers superior navigation capabilities compared to conventional catheters – for carrying the small‐scale magnetic machines (e.g., magnetic particles) close to the target site, the latter was achieved via precise magnetic navigation of magnetic particles. We anticipate that the proposed strategy will open new routes in intrathecal administration, as the conventional intrathecal therapeutic agent's delivery method – utilizing non‐magnetoresponsive drugs – is hindered by uncontrolled motion and poor targeting, potentially leading to undesirable side effects.

## 2 Results

### 2.1 Design of Magnetically Guided Microcatheter with IONPs Reservoir

We have engineered a magnetically steerable microcatheter with a reservoir position at its tip to store iron oxide nanoparticles (IONPs) as depicted in Figure 1b. This reservoir at the catheter delivers IONPs against physiological flows in the human body and releases them at the proximity of the targeted treatment site.

The outer diameter (OD) of the microcatheter was constrained to 1 mm to align with the physiological anatomy of the subarachnoid space, typically ranging from 1 to 5 mm in thickness.^[^ ^24^ ^]^ This limitation in OD poses challenges for the magnetic steerability of the microcatheter, as it restricts the total magnetic volume that can be integrated into the device. For this reason, the microcatheter's tip requires highly flexible materials to facilitate small bending radii necessary for effective magnetic steering. Conversely, the proximal end of the microcatheter necessitates stiff materials to prevent buckling during its advancement along the spinal subarachnoid space. To address these considerations, we designed the microcatheter with a rigid proximal catheter body featuring an inner lumen composed of a polytetrafluoroethylene (PTFE) liner tube surrounded by a spring, enclosed within an outer shell made of Pebax elastomer. The highly flexible catheter tip consists of a commercially available silicone tube adorned with a total of 11 neodymium magnets to facilitate magnetic steerability. A membrane separates the catheter body and the tip to retain the IONPs within the reservoir (inset in Figure 1c). The microcatheter's small OD allows for its insertion through an 18 G Tuohy needle, as illustrated in Figure 1c. Figure 1d displays a reservoir fully loaded with IONPs. The total volume of stored IONPs solution, that is, 20 µl for a 15‐mm‐long reservoir, can be ejected when a connected syringe flushes water through the membrane.

The design of the catheter tip was refined to facilitate the release of IONPs to the target against the attraction of the ring magnets attached to the catheter for magnetic steering. Numerical simulations were used to predict the trajectories of IONPs, for identifying the design that ensures the effective expulsion of the majority of particle aggregates (vide infra). For this purpose, we developed an axisymmetric simulation model placing the catheter tip at the center of a capillary tube (3 mm in diameter, Figure S1, Supporting Information) for studying the performance of three different catheter designs having short, medium, and long tip designs, with a magnet‐to‐tip distance 1, 2, and 5 mm, respectively. Numerical simulations demonstrated that the undesired attraction of the ejected magnetic particle aggregates by the ring magnets, placed at the catheter tip, can be avoided by increasing the ring magnet‐to‐tip distance (Figure 1e–g). In contrast to the short tip design (Figure 1e), which unfortunately attracts the IONP aggregates ranging from 1 to 100 µm in diameter, the medium tip design (Figure 1f) effectively circumvents this issue, not attracting aggregates with sizes between 1 and 10 µm. However, it still attracts aggregates of 100 µm. Despite the superior performance of the long tip design (Figure 1g) in minimizing undesired magnetic attraction, designs with shorter tips are more desirable due to their ability to enhance the maneuverability of the magnetic catheter. Therefore, to identify the optimal design, we performed further numerical simulations.

First, we investigated the hydrodynamic flows within and surrounding the catheter resulting from an ejection stream (e.g., 200 µl s^−1^). As seen in Figure S2 (Supporting Information), the contraction and expansion zones that are created by the attached ring magnets over the reservoir lead to the formation of a jetting stream (high velocity) and vortices (fluid recirculation) both within the catheter lumen and downstream at the lumen tip (i.e., capillary tube domain). The combined effects of these complex hydrodynamic flow fields and the magnetic forces significantly influence the trajectories of IONPs during ejection (Figure S3, Supporting Information). As the superparamagnetic IONPs can form agglomerates of different sizes under the effect of high shear stresses^[^ ^25^, ^26^ ^]^ (e.g., during ejection) and magnetic fields^[^ ^27^, ^28^, ^29^ ^]^ (induced by either the permanent ring magnets or the external fields applied by eMNS), we assumed spherical particle aggregates with a uniform size distribution ranging from 0.1 to 100 µm for the simulations. Note that the largest aggregate size was assumed to be 100 µm because the smallest geometrical section within the catheter lumen is 200 µm. After optimizing the simulation parameters (e.g., mesh size and incremental time‐step, Figures S4–S6, Supporting Information), we performed a parametric set of simulations (see Table S1, Supporting Information) to identify the optimal catheter tip design for various ejection flow rates, ranging from 10 to 200 µl s^−1^ (Figures S7–S11, Supporting Information). Note that since the classical injection during spinal anesthesia can be clinically performed at higher flow rates (reaching up to 300 µl s^−1^) and for considerably longer exposure times (e.g., tens of seconds),^[^ ^30^, ^31^, ^32^, ^33^, ^34^ ^]^ the selected ejection flow rate and time range can be considered as a safe value for biological tissues. To compare the different designs (i.e., short, medium, and long tip designs), we evaluated the percentage of the particle aggregates that were effectively ejected from the catheter tip by the time the entire volume of the solution in the reservoir had been ejected (Table S2, Supporting Information). Notably, as the ejection flow rate increased from 10 to 200 µl s^−1^, the percentage of ejected particle aggregates rose from ca. 60% to 84.6%, 86.5%, and 81.9% for the short, medium, and long tip designs, respectively. This key finding underscores the superior performance of the medium tip design, which achieved a significantly high ejection rate of up to 86.5% of particle aggregates from the reservoir, highlighting its effectiveness for particle aggregates ejection. Hence, we concluded that the medium tip design, with a magnet‐to‐tip distance of 2 mm, ideally achieves both effective particle aggregates ejection and enhanced catheter steerability (vide infra, **Section** **2.4**).

### 2.2 IONPs Ejection from the Microcatheter's Reservoir

**Figure 2:**
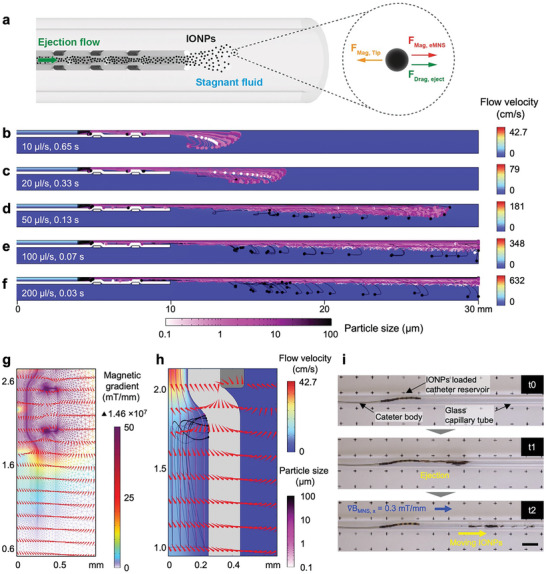
Numerical simulations and experimental results of particle aggregates ejection under stopped‐flow conditions. a) Schematic representation of particle aggregates ejection inside the capillary tube. b–f) Parametric study to investigate the ejection of aggregates from the reservoir of the catheter tip using different ejection flow rates (i.e., 10, 20, 50, 100, and 200 µl s^−1^ from b to f, respectively). The velocity maps and aggregates trajectories snapshots are shown at the time‐points immediately after the full‐ejection of the reservoir, thus at different moments depending on the imposed ejection flow rate. g) Surface map and flux lines of magnetic field gradients formed around the permanent ring magnet. h) Flux lines of magnetic field gradients affecting the particle aggregates trajectories inside the catheter. i) Experimental results of stopped‐flow ejection of IONPs inside the capillary tube and their magnetic control under the effect of magnetic field gradient (*𝛻B_MNS, x_* = 0.3 mT mm^−1^). Note that ejected IONPs are navigated inside the capillary tube using external magnetic control. (Scale bar: 10 mm).

Besides optimizing the catheter design, the parametric study provides detailed insights on the ejection of IONPs at different ejection flow conditions. Based on this insight, we turned our attention to the ejection performance of the IONPs at flow rates ranging from 10 to 200 µl s^−1^, under 30 mT of external magnetic field (to magnetize the superparamagnetic IONPs) within a stagnant fluid domain (i.e., ejection of IONPs into stopped‐flow condition, **Figure** **2**a). Note that although our catheter was designed for spinal cord applications, it can also be employed as a delivery assistant in the vasculature system, where stopped‐flow conditions could, for example, be clinically achieved by the utilization of a balloon catheter. Figure 2b–f and Table S2 (Supporting Information) show that higher ejection flow rates lead to an increase in the number of effectively ejected particle aggregates, as well as a larger longitudinal spread of the ejected particle aggregates due to strong jetting streams. Moreover, at high ejection flow rates (≥50 µl s^−1^), larger aggregates (≥100 µm) are effectively ejected from the catheter reservoir. This is an important consideration, as larger aggregates require lower magnetic field gradients for effective guidance by external magnetic field control (see **Section** **2.3**).

At lower ejection flow rates (i.e., 10 µl s^−1^), larger aggregates (10–100 µm) are attracted to the permanent magnets due to the magnetic field gradients induced around them (Figure 2g,h). The larger aggregates, possessing higher magnetic moments and experiencing lower velocities (e.g., lower ejection flow rates at the onset of the ejection ramp‐up, and the aggregates moving closer to walls of the magnet due to no‐slip boundary conditions) encounter reduced drag force. Consequently, the motion of IONPs is predominantly influenced by magnetic forces, leading to the attraction of aggregates towards the permanent magnets and thereby bending their trajectories. Therefore, by controlling the ejection flow rates, it is possible to alter the balance between the hydrodynamic and magnetic forces, thus achieving more effective ejection of particle aggregates from the catheter reservoir.

Following the numerical simulations and using the microcatheter, we experimentally demonstrated both the ejection of IONPs and their magnetic swarm motion in the stagnant fluid (stopped‐flow conditions) (Figure 2i). The microcatheter, loaded with IONPs, was introduced into a capillary tube (3 mm in diameter) filled with water (*t_0_* in Figure 2i). The IONPs were effectively ejected up to a distance of 20 mm from the catheter tip (*t_1_* in Figure 2i), a result consistent with the numerical simulation prediction for a 50 µl s^−1^ ejection flow rate (Figure 2d). Moreover, the released nanoparticle swarm was successfully navigated using external magnetic field control (*B_MNS_* = 30 mT and 𝛻*B_MNS,x_* = 0.3 mT mm^−1^ in the direction of release, *t_2_* in Figure 2i). Numerical simulation and experiments were performed in pure water, as the composition and physical properties are similar to pure water.^[^ ^35^, ^36^ ^]^ We believe the results would be the same in CSF.

### 2.3 IONPs Ejection and Magnetic Navigation Against External Flow

After analyzing the ejection of IONPs under stagnant flow conditions, we assessed their release against a counter‐flow mimicking the average physiological CSF flow rate of 2.5 cm s^−1^, prevailing around the target location in the subarachnoid space (C2 segment).^[^ ^37^, ^38^, ^39^ ^]^ As a starting point, no pulsatility was imposed on the counter‐flow because we aimed to study the ability of ejecting particle aggregates under general counter‐flow to identify the required ejection flow rates, magnetic gradients, and overall performance of the catheter in terms of particle aggregates ejection. Moreover, even though the pulsatility conditions could affect the individual trajectories of particle aggregates, the average (over a time period) percentage of ejected particle aggregates does not significantly differ in simulations with and without pulsatile counter‐flows.^[^ ^40^ ^]^ Nevertheless, it should be noted that the assumption of steady counter‐flow, despite being reasonable for the above‐mentioned aspects, is still a simplification, particularly for the cervical region where a characteristic pulsatile flow exists.^[^ ^41^ ^]^

**Figure 3:**
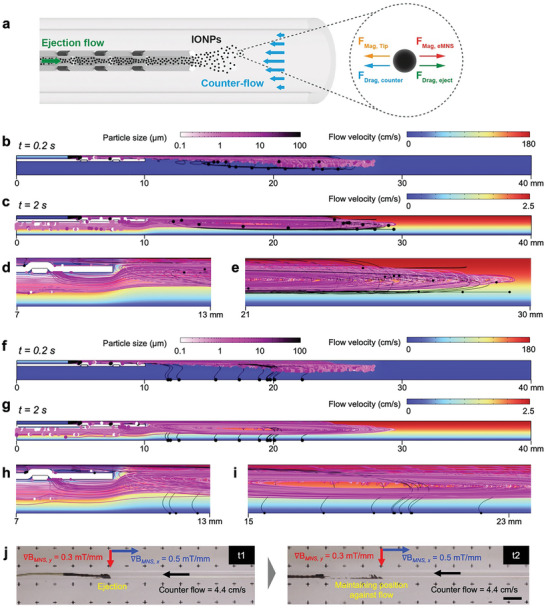
Numerical simulations and experimental results of particle aggregates ejection against the external counter‐flow under magnetic control. a) Schematic representation of particle aggregates ejection inside the capillary tube against an external counter‐flow. b–e) Simulation results that show the trajectories of aggregates ejected at a flow rate of 50 µl s^−1^ against an external counter‐flow (2.5 cm s^−1^) under the effect of axially applied magnetic field gradient, 𝛻*B_z_* = 0.3 mT mm^−1^. b,c) Presenting the velocity maps and particle aggregates trajectories at b) t = 0.2 s and c) t = 2 s respectively during and after the 1 s ejection event. d,e) Close look of the trajectories of aggregates d) away from and e) around the catheter (at t = 2 s) after the 1 s ejection event is completed, hence the trajectories of aggregates are determined by the combined effect of counter‐flow and applied magnetic field gradient. f–i) Simulation results that show the trajectories of aggregates ejected at a flow rate of 50 µl s^−1^ against an external counter‐flow (2.5 cm s^−1^) under the combined effect of axially and radially applied magnetic field gradients, respectively 𝛻*B_z_* = 0.5 mT mm^−1^ and 𝛻*B_r_* = 0.3 mT mm^−1^. f,g) Presenting the velocity maps and aggregates trajectories at f) t = 0.2 s and g) t = 2 s respectively during and after the 1 s ejection event. h,i) Close look of the trajectories of aggregates h) away from and i) around the catheter (at t = 2 s) after the 1 s ejection event is completed, hence the trajectories of aggregates are determined by the combined effect of counter‐flow and applied magnetic field gradients. j) Experimental results of counter‐flow ejection of IONPs inside the capillary tube and their magnetic control under the magnetic field gradients (𝛻*B_MNS, x_* = 0.5 mT mm^−1^ and 𝛻*B_MNS, y_* = 0.3 mT mm^−1^). Note that ejected IONPs maintain the position inside the capillary tube against the counter‐flow even at higher flow rates (4.4 cm s^−1^). (Scale bar: 10 mm).

Numerical simulations were conducted to investigate the particle aggregates release under the combined effect of this counter‐flow and an external magnetic field (**Figure** **3**a; Table S3, Supporting Information). The simulations revealed that low ejection flow rates (e.g., 10 µl s^−1^) are ineffective for releasing the particle aggregates against a counter‐flow, resulting in an undesired backflow of particle aggregates around the catheter. (Figure S12, Supporting Information). Consequently, we chose the minimum ejection flow rate as 50 µl s^−1^ to accomplish successful particle aggregates release against a counter‐flow of 2.5 cm s^−1^ (Figure 3b). Velocity maps and particle aggregates trajectories at different time points during and after the one‐second ejection event (Figure 3b–e) indicate that the particle aggregates can be effectively ejected from the microcatheter, and the particle aggerates (≥100 µm) can be successfully navigated against the 2.5 cm s^−1^ counter‐flow under an axially applied magnetic field gradient (𝛻*B_z_* = 0.3 mT mm^−1^, Figure 3e; Figures S13 and S14, Supporting Information). Notably, while the smaller particle aggregates are pushed backward by the counter‐flow, larger aggregates (≥100 µm) can be moved forward by the magnetic field gradients, overcoming hydrodynamic drag. This is because the magnetic forces acting on the larger particle aggregates can overcome the hydrodynamic drag. As the volume‐to‐surface area ratio of the aggregates increases with increasing aggregate size, the magnetic forces, which depend on the aggregate volume, become more relevant than the drag forces, which depend on the aggregate surface area. Moreover, increasing the axially applied magnetic field gradient (𝛻*B_z_*) to 0.5 mT mm^−1^ results in a longer travel distance of the particle aggregates over the same time period (e.g., 2 s). More specifically, an increase in the magnetic field gradient results in higher magnetic force acting on the particle aggregates, thereby enhancing their acceleration against the flow (Figure S15, Supporting Information). These results suggest that the particle aggregates released at the proximity of the target location could be effectively navigated into the target via magnetically controlled motion of the nanoparticle swarms.

We further exploited the effect of lower velocities near the walls of the capillary tube, due to the *no‐slip* conditions, to advance particle swarm motion control against the counter‐flow. Here, the lower fluid velocities near the walls help to overcome hydrodynamic drag with magnetic forces, facilitating precise magnetic control over the released particle swarm. Simulations under both axially and radially applied magnetic field gradients (𝛻*B_z_* = 0.5 mT mm^−1^ and 𝛻*B_r_* = 0.3 mT mm^−1^, Figure 3f–i; Figures S16 and S17, Supporting Information) showed that, at the early stage of ejection, the magnetic field gradients (𝛻*B_z_* and 𝛻*B_r_*) pulled a significant number of large particle aggregates (100 µm) from the vortices towards the wall, where hydrodynamic drag forces are relatively low. Note that, to reduce the computational cost of the simulations, particle aggregates touching the wall were assumed to remain motionless until the end of the simulation, a condition not reflecting reality. Nevertheless, their curved trajectories under combined axial and radial magnetic gradient (Figure 3f–i) and the result of magnetic motion under sole axial gradient (Figure 3b–e) suggest that these particle aggregates can still be navigated using external magnetic fields. Moreover, tailoring the ejection flow rates allows control over the target location for particle aggregates ejection. Increasing the ejection flow rates from 50 to 100 µl s^−1^ resulted in an expanded longitudinal distribution and a higher number of successfully ejected IONPs (Figures S18 and S19, Supporting Information). This finding underscores the potential for exquisite control over the targeted release of the IONPs by finely tuning the interplay between the hydrodynamic and magnetic forces (e.g., by changing ejection flow rates and magnetic field gradients, respectively).

Figure 3j shows the release of the IONPs through the microcatheter against a counter‐flow. The microcatheter, loaded with IONPs, was introduced into a capillary tube (3 mm in diameter) filled with water flowing at a speed of 4.4 cm s^−1^ opposite to the direction of ejection. Under these counter‐flow conditions, the IONPs were effectively ejected from the catheter (*t_1_* in Figure 3j) and the nanoparticle swarm was successfully maintained in a stable position against the flow by the external magnetic field (*B_MNS_* = 30 mT with 𝛻*B_MNS,x_* = 0.5 mT mm^−1^ and 𝛻*B_MNS,y_* = 0.3 mT mm^−1^), even after the microcatheter was retracted (*t_2_* in Figure 3j). Similarly, the nanoparticle swarm was also successfully maintained in a stable position in the co‐flow scenario (i.e., the fluid in the capillary tube flows in the same direction with the ejection flow) with a flow speed of 5 cm s^−1^. This stability was achieved by leveraging lateral magnetic gradients to localize the nanoparticle swarm near the wall, where local fluid velocities are slower, thereby minimizing hydrodynamic drag (Figure S20, Supporting Information).

### 2.4 Catheter Control Using Magnetic Field

**Figure 4:**
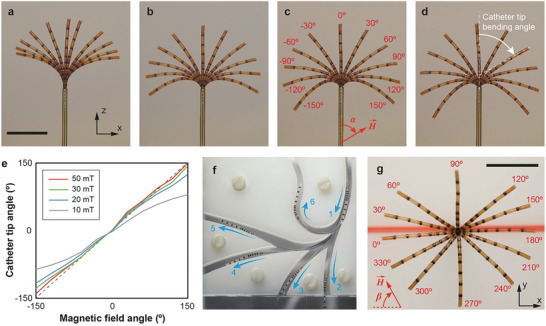
Steerability of magnetically guided microcatheter. a–d) Overlayed images (top view) of the catheter tip according to the magnetic field angle. Magnetic field angle (𝛼) varies from −150° to 150° with 30° interval (*β* = 0°), indicated in (c). Scale bar indicates 10 mm. a) 10 mT. b) 20 mT. c) 30 mT. d) 50 mT. e) Catheter tip angle change as a function of magnetic field angle. A dot line indicates the magnetic field angle equals to the catheter tip angle. f) Navigation through the curved channels having different curvature. g) Overlayed images (front view) of the catheter tip according to the magnetic field angle (*β*) from 0° to 360° (𝛼 = 90°). The magnetic field strength is 30 mT. The scale bar indicates 10 mm.

In order to characterize the steerability of the magnetic microcatheter, we evaluated the angle of the catheter tip as a function of the external magnetic field strength and angle (**Figure** **4**a–d; Videos S1–S4, Supporting Information). In the eMNS experimental setup described in Figure S21 (Supporting Information), two cameras simultaneously captured the motion of the microcatheter. The angle of the catheter tip in the x‐z plane is defined by the tangent angle of the curve at the tip (Figure 4d). As the magnetic field strength increases from 10 to 50 mT, the angle of the catheter tip accurately aligns with the magnetic field angle (𝛼) in the x‐z plane (Figure 4e). Considering that this catheter is intended for use inside the human body, we selected the magnetic field strength of 30 mT to control the microcatheter with enough steerability while ensuring its compatibility for biomedical applications. At this magnetic field strength, the microcatheter navigated along 2D pathways with various radii of curvatures (Figure 4f). In addition, motion in the x‐y plane was achieved by controlling the magnetic field angle (𝛽) in the x‐y plane with 𝛼 sets to 90° (Figure 4g; Videos S1–S4, Supporting Information). Thus, by controlling both magnetic field angles (𝛼 and 𝛽), the motion of the microcatheter can be expanded into 3D space, enabling its navigation through the complex channels of human organs, such as the spinal subarachnoid space (vide infra, Section 2.5) and the neurovascular network (Video S5, Supporting Information).

### 2.5 Proximity Injection of IONPs in the Spine Phantom

**Figure 5:**
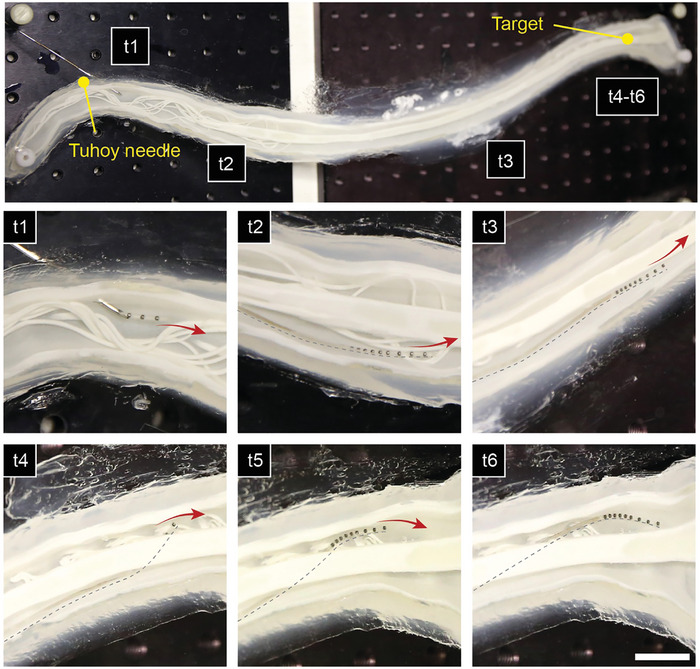
Demonstration of the microcatheter navigation through the spinal cord phantom model using eMNS. The scale bar indicates 10 mm.

We demonstrated the entire procedure of catheter advancement, magnetic navigation, injection of IONPs, and magnetic control of the IONPs within a 3D phantom model that represents the spinal subarachnoid space. The catheter was introduced into the phantom through a Tuohy needle at the lower section (e.g., lumbar vertebrae) of the spinal model and then manually advanced upwards towards the cervical vertebrae. **Figure** **5** shows the microcatheter's navigation to the target site around the C2 section, which connects the spinal cord to the brain. By using magnetic field guidance, the microcatheter was safely maneuvered around obstacles in the form of nerve rootlets (t1‐t3 in Figure 5). Note that these nerve rootles are anatomical features that can significantly affect the flow dynamics in cervical subarachnoid space during the intrathecal injection (vide infra).^[^ ^42^, ^43^ ^]^ Additionally, magnetic control facilitated the steering of the microcatheter from the anterior to the posterior side of the spine (t4‐t5 in Figure 5), enabling it to safely reach the target location (t6 in Figure 5).

**Figure 6:**
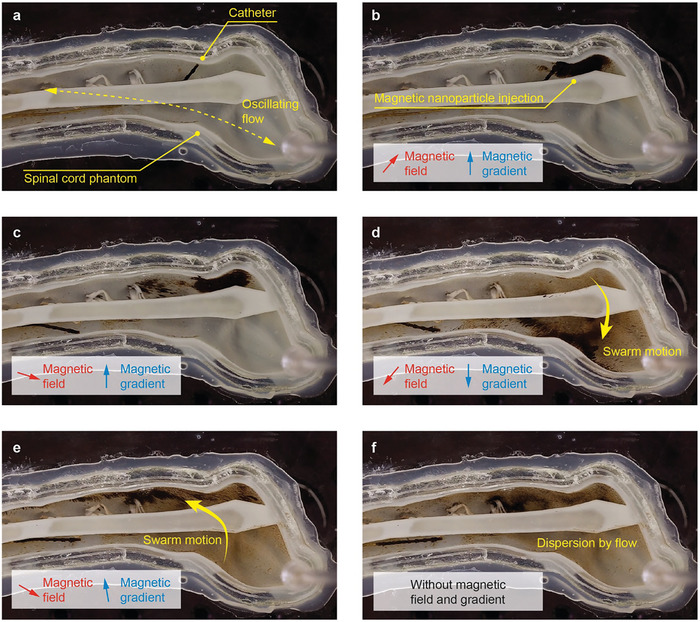
Control of magnetic nanoparticles (IONPs) using a gradient magnetic field in spinal cord phantom with oscillating flow that mimics the physiological CSF flow in the subarachnoid space. A gradient field navigates IONPs to the anterior and posterior sides of the subarachnoid space filled with water. When the magnetic field turns off, IONPs are quickly dispersed by oscillating flow.

After the catheter navigation, we demonstrated the injection and magnetic control of the IONPs at the target location under the oscillating flow conditions (2.5 cm s^−1^, altered at 1 Hz), mimicking the physiological CSF flow (**Figure** **6**). The flow was generated by a syringe pump connected to the flow inlet near the cervical vertebrae of the spinal cord phantom, and the flow rate was measured by a flowmeter connected to the outlet near the sacrum of the spinal cord phantom (Figure S22 and Video S6, Supporting Information). After injecting the IONPs along the wall of the subarachnoid space under the magnetic fields and gradients (Figure 6b), the catheter was retracted to avoid magnetic attraction of the IONPs by the ring magnets on the reservoir (Figure 6c). Subsequently, magnetic gradients were effectively used to control the IONP swarm (Figure 6d,e; Video S7, Supporting Information), minimizing their dispersion. The magnetic gradients primarily displace the IONP swarm between the anterior and posterior sides of the spine, while the magnetic fields rotate the orientation of the swarm. We noted that the orientation of the ribbon‐like IONP swarm influenced its motion under drag caused by the oscillating flow. Specifically, the ribbon‐like swarms exhibited greater fluctuations when their orientation was perpendicular to the direction of oscillating flow (Video S7, Supporting Information).

We also observed that the IONPs dispersed when the magnetic field was not applied (Figure 6f; Video S7, Supporting Information), due to the pulsatile flow field existing inside the phantom. This highlights the crucial role of the magnetic field in minimizing the dispersion of the IONP swarm, which would otherwise occur under the prevailing physiological flow conditions. Additionally, when the IONPs were injected using a needle without the application of magnetic fields or gradients, they were easily dispersed throughout the subarachnoid space by the oscillating flow. This indicates that needle injection near the lumbar vertebrae without magnetic control is inadequate for achieving targeted delivery (Video S8, Supporting Information). In summary, these demonstrations confirm that the proposed targeted delivery strategy, which includes a magnetically guided microcatheter with a reservoir and magnetic control of IONPs at the target site, efficiently delivers small‐scale magnetic devices under the circulating flow conditions through the spine.

## 3 Conclusion

We have developed an advanced magnetic microcatheter equipped with a scalable reservoir at the tip for on‐demand ejection of IONPs in close proximity to targeted release sites. Through numerical simulations and experimental studies, we established practical operating conditions that enable the microcatheter to safely release IONPs. The microcatheter‐assisted targeted delivery approach was evaluated for delivering magnetic nanoparticles at a target location within a phantom that replicates the anatomy of the spinal subarachnoid space and the CSF flow. This delivery concept can be expanded from nanoparticles to microscale devices and micro‐/nanorobot swarms through a comprehensive study of operating conditions that accommodate scaling. Moreover, advanced control over magnetic nanoparticle swarms using different magnetic fields can work in tandem with our targeted delivery approach. We anticipate that our synergistic approach will overcome challenges in implantation and delivery procedures using magnetic drug carriers, resulting in reduced dispersion and improved effectiveness in targeting.

## 4 Experimental Section

### Catheter Body

The catheter body consists of an inner lumen made from a PTFE liner tube surrounded by a spring enclosed by an outer shell made from Pebax elastomer. The spring, stretched over the entire length of the body, prevents the catheter from buckling, and keeps the catheter robust, yet flexible. The Pebax shell with 0.9 mm OD gives the catheter body the necessary stiffness and agility. Here, the catheter body was segmented into three sections of Pebax tubes that differ in stiffness ranging from 72 D, 50 D down to 35 D (Shore D Hardness) close to the tip, ensuring the highest steering capabilities, with a flexible section close to the tip, yet stiff proximal end, to sustain axial forces that are necessary to advance the catheter by pushing.

The catheter body was fabricated layer by layer, first by preparing the custom spring with 0.5 mm inner diameter (ID) made from stainless steel wire (V4a‐316L, awg42). The custom spring was created by winding said steel wire manually around a rotating PTFE‐coated rod with 0.45 mm OD. The winded wire was then pulled, released, and cut to the desired length. The spring was then laced on top of the PTFE liner (Nordson Medical, 0.44 mm OD) that is factory pre‐mounted on a metal mandrel to give it support while handling. Eventually, biocompatible thermoplastic Pebax with different stiffnesses were pulled over the spring‐liner assembly. The 35 D Pebax (Duke Extrusion, PebaSlix, 0.84 mm ID, 0.99 mm OD) tube was placed in the front with an approximate length of 50 mm, followed by the middle section with 55 D Pebax tube (Nordson Medical, 0.74 mm ID, 0.99 mm OD) with a length of 100 mm and the proximal section with 72 D Pebax (Nordson Medical, 0.74 mm ID, 0.99 mm OD). After joining the Pebax tubes edge to edge, a polyester heat‐shrink tube (Nordson Medical Inc., 1.14 mm ID) was pulled over the length of the whole assembly for a reflow lamination process where all parts were fused together at 300 °C. Finally, the heat‐shrink tube was removed and the mandrel on which the PTFE liner was factory pre‐mounted was removed to form a lumen, finalizing the preparation of the catheter body.

### Catheter Tip

The catheter tip was fabricated using a commercially available silicone tube. It includes an 11 (4 + 7)‐magnet configuration to facilitate magnetic navigation. Custom ring magnets (Hangzhou X‐Mag, N52, 0.95 mm OD – 0.65 mm ID – 0.5 mm thickness) were fitted to the silicone tube (Quosina, T2002, 50 A, 0.90 mm OD – 0.50 mm ID) and secured in place through interference fit and friction. In this magnet configuration, the front 4 magnets control the bending of the silicone tube, while the remaining 7 magnets at the back control the catheter body. A permeable membrane (Sigma–Aldrich, Teflon filter membrane, 1.5 µm) was clamped between the last two magnets positioned between the catheter body and the catheter tip within a polyester shrink tube (Nordson Medical, 1.14 mm ID). This tube effectively joined both catheter parts through reflow lamination at low temperatures, up to 200 °C, to prevent membrane melting. As IONPs are loaded into the catheter tip's lumen by aspiration, the permeable membrane is crucial to prevent IONPs from flowing backward into the catheter body. This precaution is essential during magnetic navigation to avoid magnetic agglomeration, potential catheter malfunction, and lumen clogging. The permeable membrane's pore size is a critical factor affecting the pressure required to eject fluid from the catheter tip. Consequently, porosity should be adjusted based on the application and micro‐ and nanorobot size to achieve higher ejection flow rates at reduced pressures.

### Characterization of IONPs

Commercially available IONPs (Chemicell, fluidMAG‐D 4101, size: 50–200 nm) were used as model nanorobotic swarms in experiments and were characterized using a vibrating sample magnetometer (VSM, Microsense, EZ9) to obtain magnetization curves.

### Numerical Simulations for Particle Ejection and Magnetic Attraction

Particle tracking studies were conducted using numerical simulations (COMSOL Multiphysics) to explore the dynamics of the release of the IONPs and aggregates from the catheter tip. The numerical studies allowed us to screen possible catheter designs and ejection flow rates, for identifying those resulting in the ejected IONPs being magnetically attracted back to the ring magnet at the catheter tip. The problem was simplified to the release of IONPs in a straight tubular channel under laminar flow. As the geometry of the problem is rotationally symmetric, a 2D axisymmetric simulation domain was designed (with *e_r_* and *e_z_* as plane axis derived from the cylindrical coordinate system, Figure S1, Supporting Information). The microcatheter was modeled in a cylindrical tube domain with a diameter of 3 mm to ensure the simulation domain includes the entire flow field around the catheter and its influence over the trajectories of the IONPs. The physics of the problem include the dynamics of fluid flow, the electromagnetic actuation of the magnetic particle aggregates and the tracking of their motion as the results of the various forces acting on them. These problems can be described by principles of fluid dynamics and electromagnetism acting on a magnetic particle using a finite element modeling approach (see **Details on Numerical Simulations** in Supporting Information). A one‐way approach was used to study how the external flow affects the trajectory of ejected IONPs under the influence of magnetic forces arising from the ring magnet or the eMNS (external magnetic control). To obtain the insight into the trajectories of IONPs and their aggregates, a time‐dependent solver was used (See also Supporting Information for more details).

### Release and Control of IONPs

The IONPs in fluid (Chemicell, fluidMAG‐D 4101) were loaded into the catheter tip by aspiration until the reservoir in the catheter tip was filled. The catheter was then inserted into the fluidic system through a hemostasis valve and was advanced into the center of the test tube which was placed in the CardioMag (a large‐scale eight‐electromagnet eMNS, maximum magnetic field strength: 80 mT, workspace: 20 cm × 20 cm × 20 cm). The catheter was then magnetically aligned in the test tube with varying magnetic field strengths in the range of 30–50 mT. The IONPs were ejected from the catheter by manual injection of water through the microcatheter. Finally, the IONPs were controlled by magnetic field gradients ranging from 0.2 to 0.5 mT mm^−1^ at various directions.

### Catheter Tip Characterization

The steering angles of the catheter tip were assessed in open space within the CardioMag, the in‐house eMNS. This was done by securing the catheter body inside an 18 G needle and rotating the magnetic field around the catheter's longitudinal axis at various field strengths, ranging from 10 to 50 mT.

### Preparation of 3D Phantom of the Spinal Cord

The maneuverability of the fabricated microcatheter was assessed in a reconstructed anatomical phantom of the spinal subarachnoid space (SSS). To create this 3D model of the SSS, an open‐source model was utilized from the research project by Sass et al.^[^ ^39^ ^]^ Their model, based on a subject‐specific high‐resolution anatomic MRI of a 23‐year‐old female, depicted the 3D fluid‐filled geometry with various levels of anatomic complexity. This included dorsal and ventral nerve‐rootlets of the spinal cord, the coccygeal nerve, and the dural wall. Notably, some features such as denticulate ligaments, the ventral median fissure, and the complex networks of tiny blood vessels and arachnoid trabeculae were omitted for simplification by Sass et al.^[^ ^39^ ^]^ The provided STL (Standard Tessellation Language) files underwent editing using Blender and Siemens NX 12. The model was sectioned along the long axis and modified to create a shell suitable for 3D printing with translucent flexible resin (Stratasys, Objet500 connex 3, VeroClear‐Tango resin blend). To fit within the 3D printer's workspace, the model was scaled down by 10%, resulting in a printed phantom with a final length of 557 mm. After printing, support material removal involved immersion in a 10 mg mL^−1^ NaOH solution (Sigma–Aldrich) over several days, followed by cleaning with fresh water and air drying. Subsequently, the phantom was carefully attached with super glue (Loctite, 401) onto a pre‐cut acrylic sheet, and the edges were sealed with silicon glue (Dow Corning, 732). The pulsation flow in the spinal cord phantom was achieved using the syringe pumps (neMESYS 290 N, Cetoni GmbH, Germany).

### Navigation of Microcatheter Through the Spinal Subarachnoid Space

The maneuverability was tested in the CardioMag, where the phantom was mounted into. The phantom was accessed with an 18 G Tuohy needle in the lower sections of the spine (roughly S3 – S4) and the catheter was guided through the phantom by steering the tip with a magnetic field of 30–50 mT to yield necessary steering angles and bending radii.

## Conflict of Interest

The authors declare no conflict of interest.

## Supporting information

Supporting Information

Supplemental Video 1

Supplemental Video 2

Supplemental Video 3

Supplemental Video 4

Supplemental Video 5

Supplemental Video 6

Supplemental Video 7

Supplemental Video 8

## Data Availability

The data that support the findings of this study are available from the corresponding author upon reasonable request.
