# Supplementary material for: Magnetically Guided Microcatheter for Targeted Injection of Magnetic Particle Swarms
Source: Adv Sci (Weinh). 2024 Aug 9;11(38):2404061. doi: 10.1002/advs.202404061 (PMC11481240; doi:10.1002/advs.202404061)
Supplement: Supplementary file 1 — Supporting Information [file ADVS-11-2404061-s008.docx]

Supporting Information

Magnetically Guided Microcatheter for Targeted Injection of Magnetic Particle Swarms

Harun Torlakcik, Semih Sevim*, Pedro Alves, Michael Mattmann, Joaquim Llacer-Wintle, Maria Pinto, Rosa Moreira, Andreas D. Flouris, Fabian C. Landers, Xiang-Zhong Chen, Josep Puigmartí-Luis, Quentin Boehler, Tiago Sotto Mayor*, Minsoo Kim*, Bradley J. Nelson, Salvador Pané*

Table of Contents

Details on the numerical simulations 2

Supporting Figures 6

Supporting Tables 29

Supporting References 30

Details on the numerical simulations

***Laminar flow.*** To investigate the ejection of the IONPs through a catheter, a computational fluid dynamics approach based on the finite element method was used to compute the flow field in the simulation domain. The flow regime is laminar given the Reynolds numbers associated with the ejection flow rate (i.e., 10-200 µl s^-1^, 25 < Re < 510) and with the average velocity of the counter-flow (i.e., 2.5 cm s^-1^, Re = 75). The velocity and pressure in the domain were calculated by coupling the continuity and Navier-Stokes equations for incompressible Newtonian fluids, given by

|  | $\nabla\vec{U}=0$ | (1) |
| --- | --- | --- |

|  | $\frac{\partial\vec{U}}{\partial t}+\vec{U}\left( \nabla\cdot\vec{U} \right)=-\frac{1}{\rho_{F}}\nabla P+\upsilon\nabla^{2}\vec{U}$ | (2) |
| --- | --- | --- |

where $\vec{U}$ is the velocity vector, $\rho_{F}$ is the fluid density, $P$ is the pressure, $\upsilon$ is the kinematic viscosity, $\nabla$ and $\nabla^{2}$ are the nabla and the Laplacian operators.

***Magnetic fields******.*** Gauss’s law for magnetic fields given by

|  | $\nabla\cdot\vec{B}=0$ | (3) |
| --- | --- | --- |

with the scalar magnetic potential as the dependent variable given by

|  | $\vec{H}=-\nabla\Psi$ | (4) |
| --- | --- | --- |

where $\vec{B}$ is the magnetic flux density, $\vec{H}$ is the magnetic field strength, and $\Psi$ is the magnetic scaler potential, was used to calculate the magnetic fields in the domain, from which the associated magnetic gradients and magnetic forces can be derived. The ring magnets at the catheter tip were modeled as axially magnetized permanent magnets with a remanent flux density of 1.45 T (according to the data provided by the supplier Hangzhou X-Mag on the N52 magnets, **Figure S3**). A constant magnetic flux density of 30 mT was also considered in the $e_{z}$ direction (**Figure S1**), to account for the magnetic field generated by the electromagnetic navigation system (eMNS) to magnetize the IONPs (**Figure S3**). In some simulations, a magnetic field gradient was also considered to enable the magnetic manipulation of the IONPs aggregates (see below).

***Particle tracing.*** Single IONPs can agglomerate to form aggregates of different sizes, in response to the applied magnetic fields as well as high shear stresses due to the ejection flow. ^[1–4]^ To account for this in the numerical simulations, we considered uniformly aggregated IONPs of spherical shape with discrete particle size of 0.1 µm to 100 µm, following a logarithmic distribution. The velocity and trajectory of each IONP aggregate was computed by solving Newton’s second law of motion given by

|  | $\frac{\partial(m_{p}\cdot\vec{v})}{\partial t}=\sum\vec{F_{t}}$ | (5) |
| --- | --- | --- |

where $m_{p}$ is the aggregate mass, $\vec{v}$ is the velocity, and $\sum\vec{F_{t}}$ is the sum of forces acting on the aggregate along its trajectory. We considered the IONPs aggregates to be affected by magnetic forces and by drag, with other forces assumed to be negligible given the size of the aggregates (> 0.1 µm). The drag force was computed by

|  | $\vec{F_{D}}=m_{p}\frac{\left( \vec{u}-\vec{v} \right)}{\tau_{p}}$ | (6) |
| --- | --- | --- |

where $\vec{u}$ is the fluid velocity, $\vec{v}$ is the aggregate velocity, and $\tau_{p}$ is the aggregate relaxation time.

The magnetic force ($\vec{F_{M}}$), induced on the aggregate by the ring magnets at the tip of the catheter and by the eMNS, can be defined considering the properties of a quasi-static magnetic field and Maxwell's equation

|  | $\vec{F_{M}}=m_{p}\cdot\nabla\vec{B}\cdot\vec{M}= m_{p}\cdot\left[ \begin{matrix} \frac{\partial B_{r}}{\partial r} & \frac{\partial B_{z}}{\partial r} \\ \frac{\partial B_{r}}{\partial z} & \frac{\partial B_{z}}{\partial z} \end{matrix} \right]\cdot\left[ \begin{matrix} M_{r} \\ M_{z} \end{matrix} \right]$ | (7) |
| --- | --- | --- |

where $\vec{M}$ is the IONPs magnetization (obtained from Figure S3a, unit: emu g^-1^) and $\nabla\vec{B}$ is the applied magnetic gradient. The IONP mass was computed based on their density ($\rho_{P}$ = 5.20 g cm^-3^) and size (i.e., 0.1-100 µm). The magnetization of the IONPs was computed based on the applied global static magnetic field and on the intrinsic magnetic properties of the IONPs, obtained from the M-H hysteresis curves through the VSM measurements (**Figure S3a**). The magnetization vector $\vec{M}$ has only one spatial component, as the magnetic moment aligns itself instantaneously with the external magnetic field. A vector field $\vec{B}$ in a current free space can be assumed to have a zero trace and a symmetric gradient matrix.

***Modelling approach, discretization and boundary conditions.*** The catheter placed in the lumen of a capillary was modelled using an axisymmetric 2D geometry, with the symmetry axis matching the center of the catheter (**Figure S1-S2).** The domain was meshed using unstructured elements, with inflation layers near the walls to accurately capture the gradients in the flow velocity near the walls. Mesh independence tests were conducted using three meshes with ~25k, ~100k, and ~700k elements (**Figure S4**), from which the flow profile and the magnetic field were compared at different domain locations (**Figure S5a-b**). This shows that the mesh with ~100k elements is adequate for capturing the flow velocity profiles and the magnetic fields across the simulation domain. Thus, we used it in all the simulation in this work.

The fluid properties were considered to be those of water (i.e., density: 1000 kg m^-3^; viscosity: 0.001 Pa·s), both inside the catheter and in the lumen of the capillary (given that the cerebrospinal fluid (CSF) is 99% water,^[5]^ the properties of the counter-flow were assumed to be those of water).

The catheter ejection flow was imposed at the bottom of the domain (**Figure S1-S2**) and assumed to be laminar and fully developed (Equation (8)), with different flow rates (10-200 µl s^-1^).

|  | $u\left( r \right)=u_{max}\left( 1-\frac{r^{2}}{R^{2}} \right)$ | (8) |
| --- | --- | --- |

To mimic the ejection procedure, the ejection flow was applied using a smooth pulse (total duration of 1 s with transition zone of 0.01 s at the beginning and at the end of the pulse), after which the ejection flow rate was set to zero until the end of the simulation. The counter-flow at the capillary lumen was imposed at the top of the domain (**Figure S1-S2)** for the whole duration of the simulation and was assumed to be laminar and fully developed with an maximum velocity of 2.5 cm s^-1^ (in line with the average maximum velocity of the physiological CSF flow at the C2 segment of the subarachnoid space reported in the literature).^[6,7]^ The outlet was defined at the bottom edge of the domain (**Figure S1-S2)** with zero-gauge pressure. No-slip condition was assumed at the walls of the catheter, ring magnets and capillary (**Figure S1-S2**). To account for the IONPs aggregates already inside the catheter lumen at the beginning of the ejection process (t = 0 s), five IONPs aggregates of the four different sizes considered (0.1, 1, 10, and 100 µm) were homogeneously distributed along the sections 1, 2, and 3 (**Figure S1-S2**).

During the ejection process, five aggregates of each size were released from the section 3 at ten different time instants, in steps ($t_{{ejection}_{step}}$) calculated as a function of the volume of the catheter lumen ($V_{C}$) and the ejection flow rate ($Q_{f})$, i.e., $t_{{ejection}_{step}}=V_{C}/Q_{f}/10$ and $t_{{ejection}_{End}}=V_{C}/Q_{f}$.

Regarding the discretization in time, the flow profile (**Figure S5c**) at different domain locations for  t = 0.02 s and the trajectory of aggregates (**Figure S5d**) were compared for three different time-steps, namely ${2.5\times10}^{-3}\cdot\left( \frac{Q_{f}^{max}}{Q_{f}} \right)$, ${2.5\times10}^{-5}\cdot\left( \frac{Q_{f}^{max}}{Q_{f}} \right)$, and ${2.5\times10}^{-6}\cdot\left( \frac{Q_{f}^{max}}{Q_{f}} \right)$, with $Q_{f}^{max}$ = 200 µl s^-1^. The results show that the time-step of $\Delta t={2.5\times10}^{-5}\cdot\left( \frac{Q_{f}^{max}}{Q_{f}} \right)$ could adequately capture the flow profiles and the aggregate trajectories, with sorter steps yielding similar results (**Figure S5c-d).** This time-step was used for the time-dependent flow calculations (Equation (2)) and for the iteration of the particle motion (Equation (5)).

***Solver and convergence criteria.*** A fully coupled right-linear system based on the MUMPS (steady-state) and on the PARDISO (time-dependent) solvers was used with second-order discretization of the magnetic scalar potential and flow velocity, and first order discretization of the pressure. Convergence was assumed when the relative tolerance was less than 10^-5^ and absolute tolerance less than 0.05, with stricter criteria producing similar results.

***Validation.*** To validate the laminar flow profiles imposed at the catheter and capillary inlets, the numerical flow predictions at z = 4 mm (close to the catheter inlet) and z = 37.5 mm (close to the capillary inlet) were compared in **Figure S5e** with the analytical solution of Equation (8). The good agreement between the numerical predictions and the analytical solution shows that the obtained flow profiles are accurately captured and that the present modelling approach can be used to predict the flow developing inside the catheter and the capillary.

**Supporting Figures**


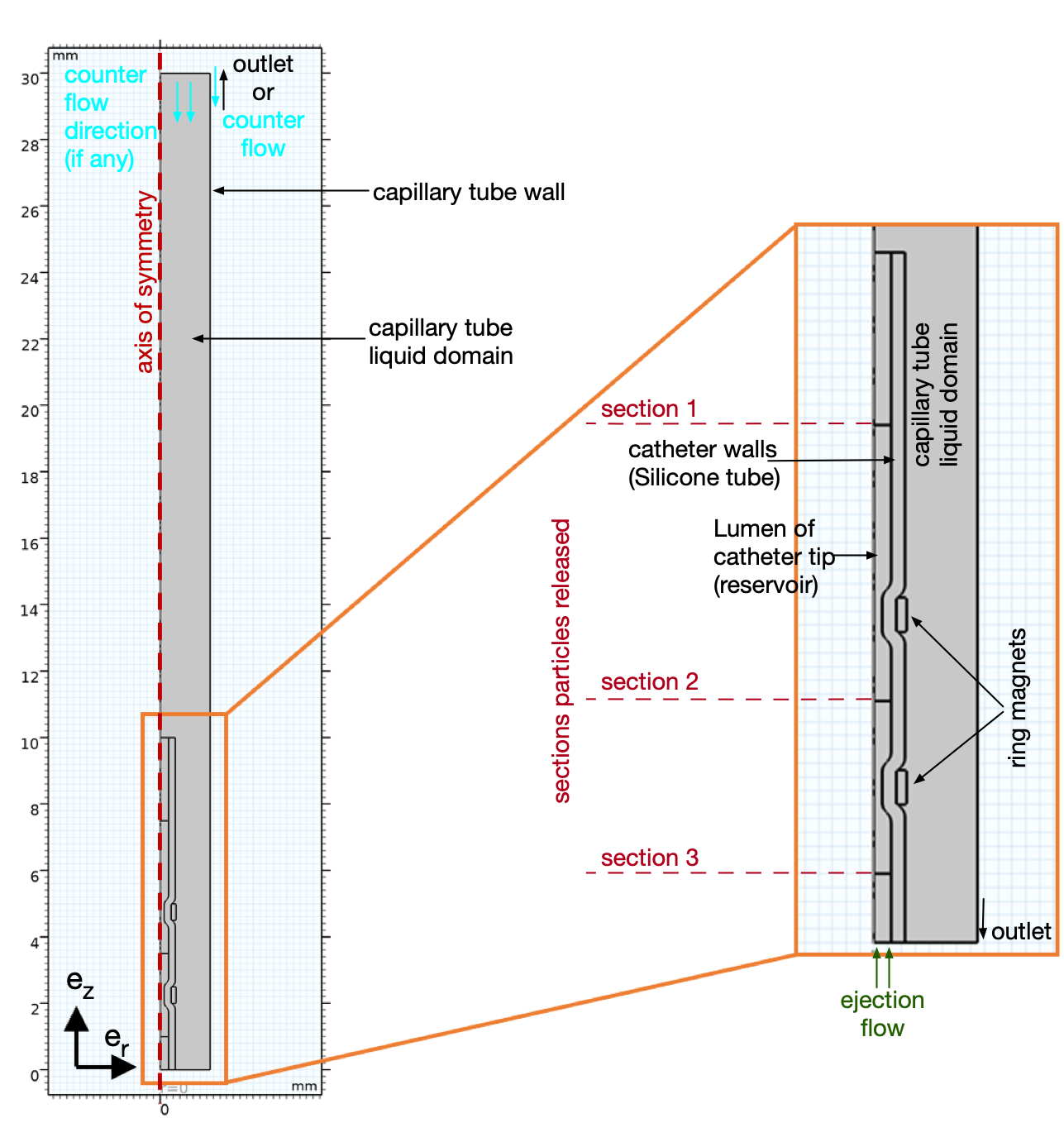


Figure S1. Illustration of simulation domain and boundaries.


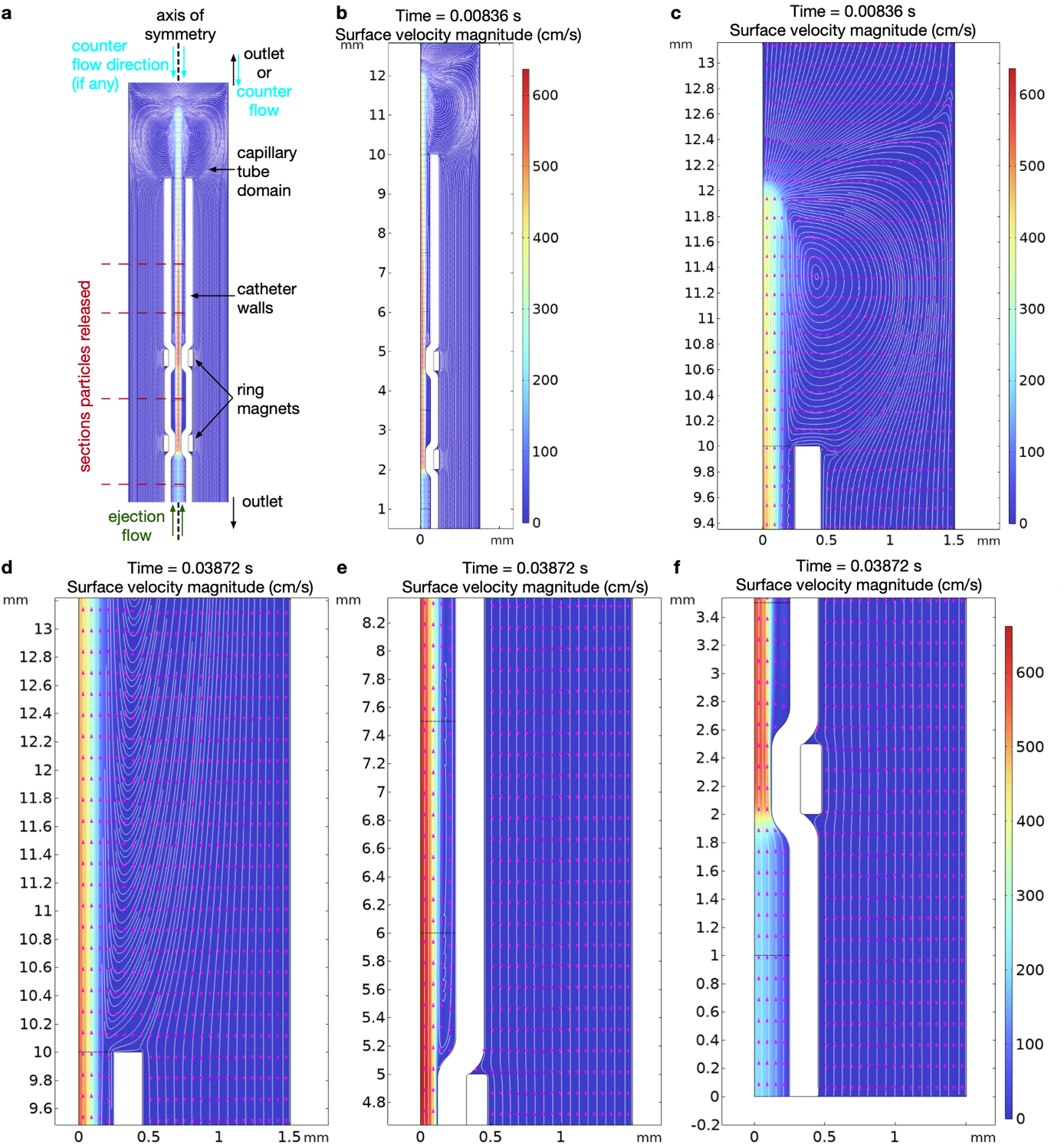


Figure S2. Hydrodynamic flow simulations of ejection at 200 µl s^-1^. (a) Axisymmetric simulation model. (b-c) Surface velocity maps and streamlines during an early time-step (t = 0.00836 s) of the ejection showing the formation of jetting stream and vortices outside the catheter. (d-f) Surface velocity maps and streamlines during a later time-step (t = 0.03872 s) showing the formation of jetting stream and vortices inside the catheter due to expansion and contraction zones resulted by press-fitted ring magnets.


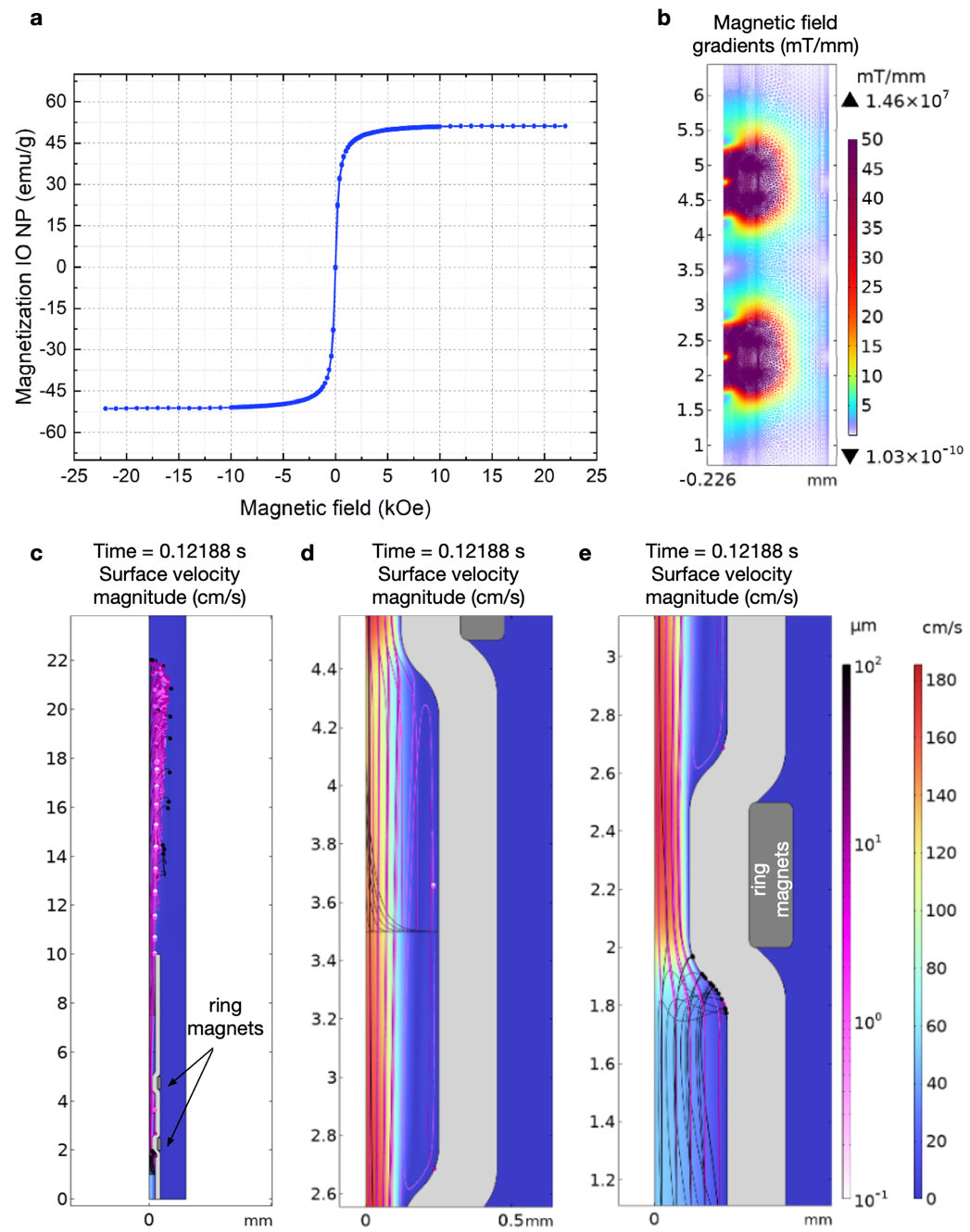


Figure S3. Magnetic attraction of superparamagnetic IONPs due to the ring magnets localized over the catheter. (a) VSM results showing the magnetization loop of commercial superparamagnetic IONPs. (b) Surface map of magnetic field gradients formed around the permanent ring magnets. (c-e) Surface velocity map and particle aggregates trajectories at t = 0.00836 s of the ejection (at 50 µl s^-1^), showing the trajectories of (c) ejected aggregates that are color-coded based on their sizes (ranging from 0.1 µm to 100 µm), (d) aggregates trapped in the vortices formed within the catheter, and (e) aggregates attracted by permanent ring magnets. Note that on bigger aggregates (10-100 µm), magnetic forces are more dominant than the hydrodynamic forces especially at smaller velocities (e.g., early step of ejection, smaller ejection flow rates, and slower aggregates close to the walls of magnet due to *no-slip* boundary conditions) resulting in the attraction of aggregates towards permanent magnets, hence bending their trajectories.


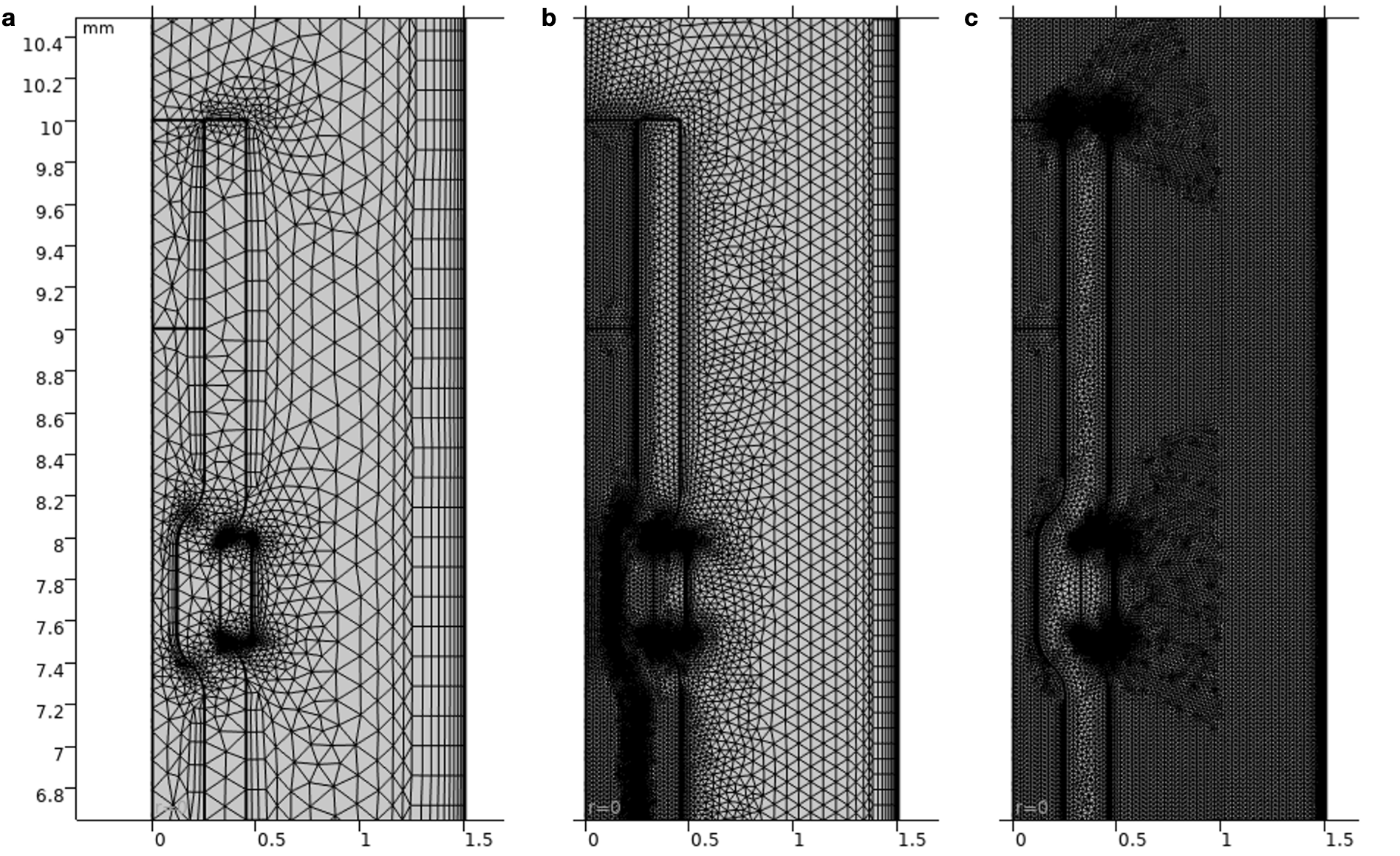


Figure S4. Unstructured meshes of increasing density, used for the mesh independence tests: (a) mesh with ~25k elements, (b) mesh with ~100k elements, and (c) mesh with ~700k elements. Mesh (b) with ~100k elements was shown to be adequate for capturing the flow velocity profiles and the magnetic fields.


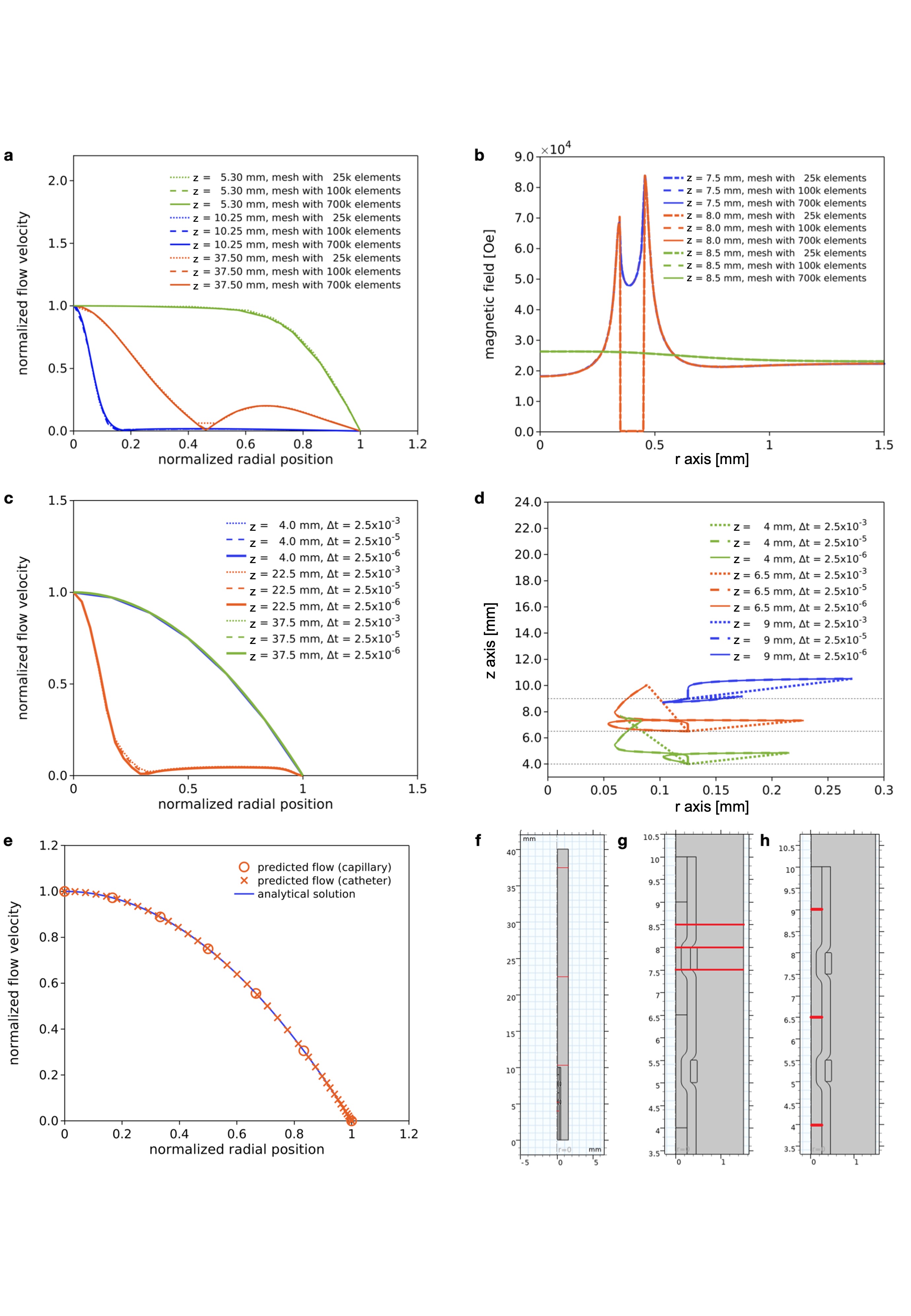


**Figure S5.** Mesh and time-step independence tests, and comparison of the predicted flows in the catheter and the capillary against analytical solutions, for an ejection flow rate of 100 µl s^-1^ and counter flow of 2.5 cm s^-1^. (a) Normalized flow profiles at three different locations (z = 5.30, 10.25, and 37.50 mm) obtained using three different meshes (25k, 100k, and 700k elements). (b) Magnetic field at three different locations (z = 7.5, 8.0, and 8.5 mm) obtained using three different meshes (25k, 100k, and 700k elements). (c) Normalized flow profiles at three different locations (z = 4.0, 22.5, and 37.5 mm) at t = 0.02 s, obtained using three different time steps (2.5×10^-3^, 2.5×10^-5^, and 2.5×10^-6^). (d) Particle aggregates trajectories at three different initial positions (z = 4.0, 6.5, and 9.0 mm) obtained using three different time steps (2.5×10^-3^, 2.5×10^-5^, and 2.5×10^-6^). (e) Comparison of the flow profile predicted by the numerical simulations and that obtained with an analytical solution for laminar flows. (f) Catheter and capillary positions (z = 4.0, 5.3, 10.25, 22.5, and 37.5 mm) for plotting (a) and (c). (g) Catheter and capillary positions (z = 7.5, 8.0, and 8.5 mm) for plotting (b). (h) Catheter positions (z = 4.0, 6.5, and 9.0 mm) f for plotting (d). The results of (a) and (b) show that the mesh with 100k elements provides mesh-independent flow profiles and magnetic fields. The results of (c) and (d) show that a time step of $\Delta t={2.5\times10}^{-5}\cdot\left( {Q_{f}^{max}}/{Q_{f}} \right)$ allows to capture the flow profiles and the trajectories. Note that the fraction (${Q_{f}^{max}}/{Q_{f}}$) is not shown in the figure legend for the sake brevity. Finally, the results of (e) show that the velocity profiles in the catheter and in the capillary perfectly match those obtained with an analytical solution for laminar flows.


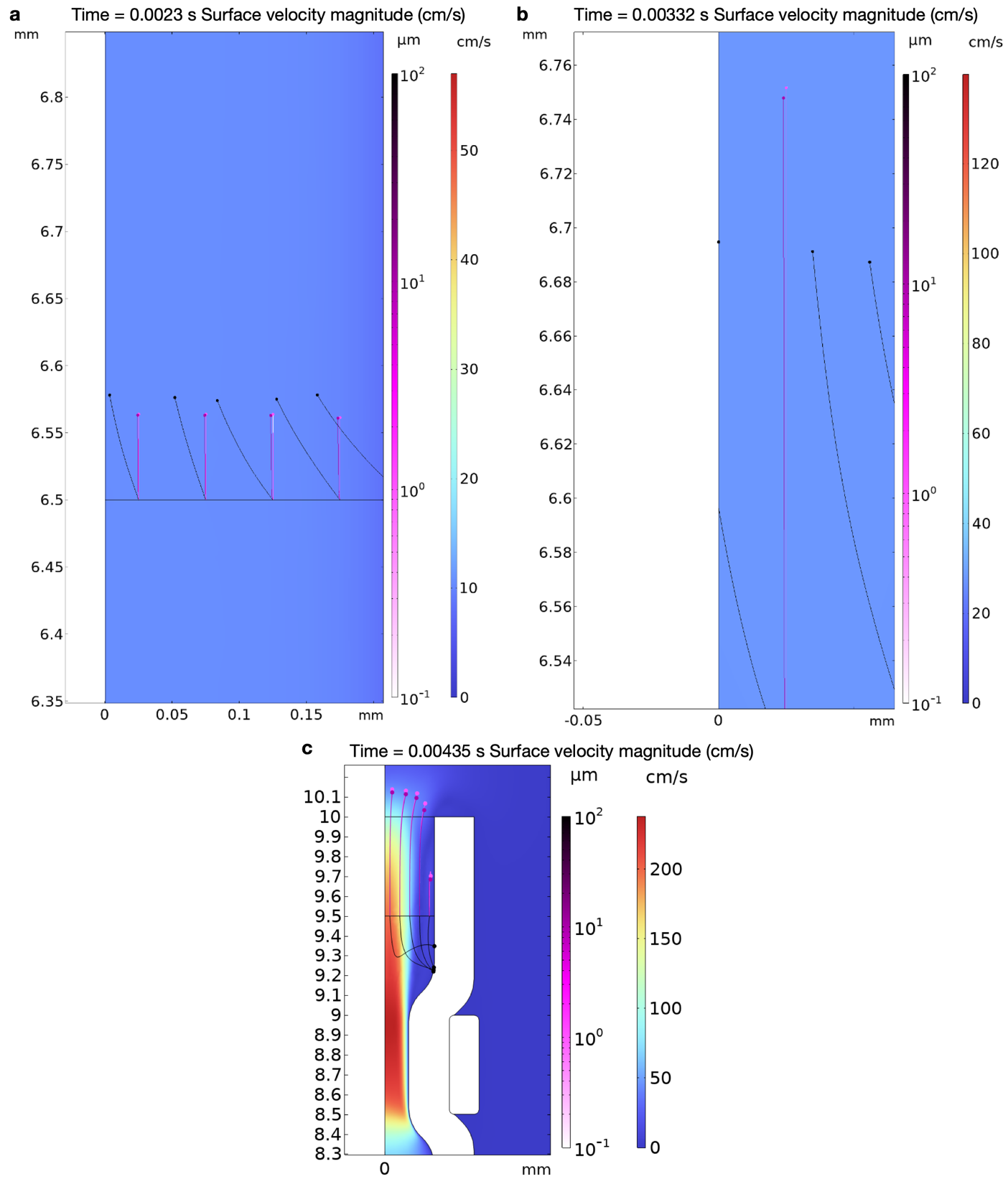


Figure S6. Boundary condition (BC) assumed at symmetry axis and time increment optimization in transient numerical simulations. (a-b) Surface velocity maps and particle aggregates trajectories around the axis of symmetry respectively at t = 0.0023 s and t = 0.00332 s of the ejection. To avoid the undesired disappearance of the aggregates at the symmetry axis and keep them in the simulation domain, a negligible x-axis velocity is assigned if the aggregates touch the symmetry axis. Note that this BC assumption allows for estimating a reliable statistic regarding the aggregates tracking (i.e., the number of aggregates ejected from the catheter tip) whilst causing a negligible effect in their trajectories. (c) Surface velocity map and aggregates trajectories around the tip of the catheter. As a result of optimized incremental time-steps, numerical simulations demonstrate smooth trajectories for aggregates even at an early time-step (t = 0.00435 s) of ejection with high flow rates.


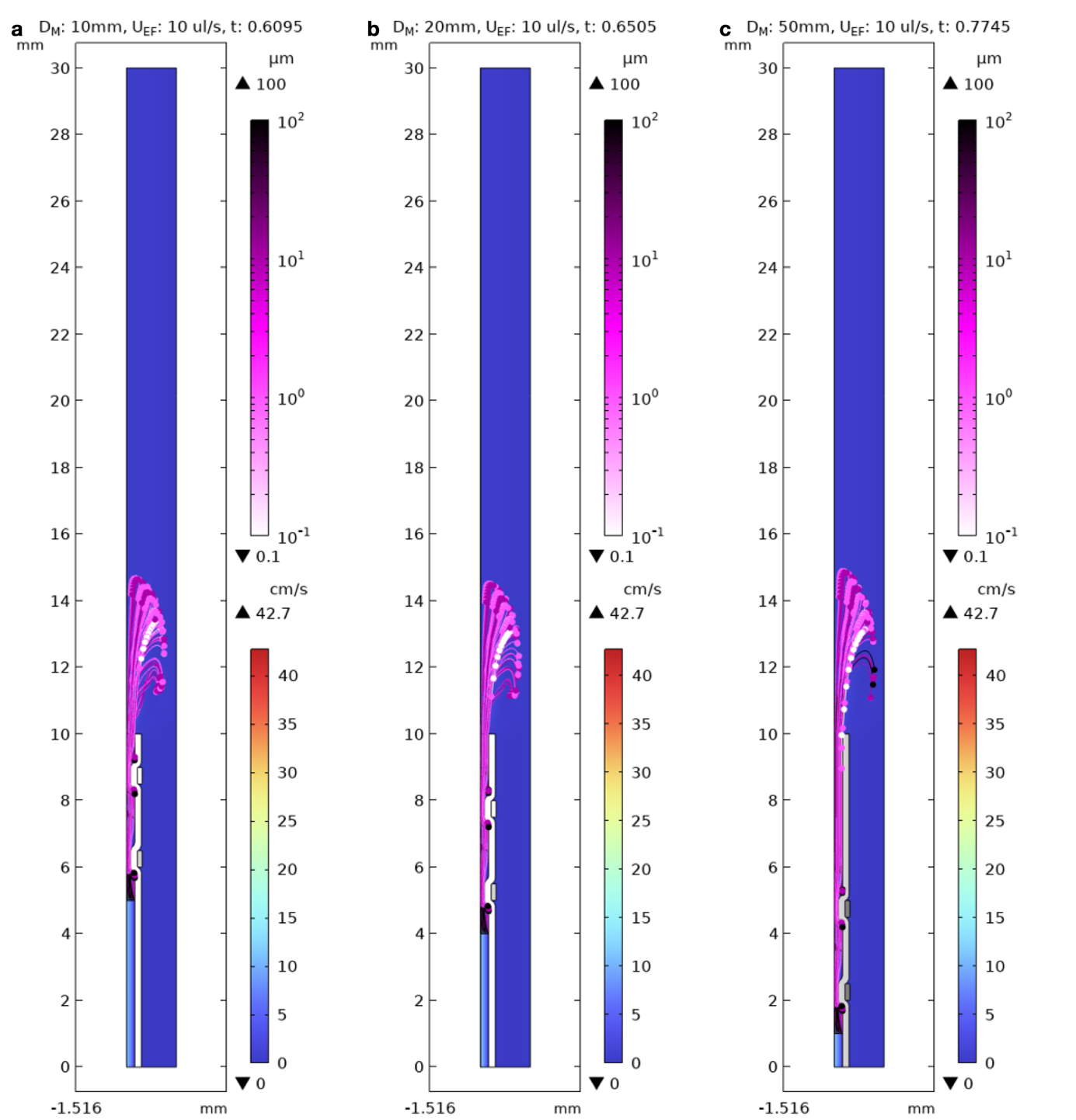


Figure S7. Parametric numerical simulations to optimize the catheter design (i.e., distance from the ring magnet to the catheter tip) for effectively ejecting (at an ejection flow rate of 10 µl s^-1^) the particles from the reservoir of the catheter tip. (a-c) Surface velocity maps and particles trajectories at the time-point when the full-ejection of the reservoir is completed for various designs having a catheter tip to magnet distance of (a) 1 mm, (b) 2 mm, and (c) 5 mm, respectively.


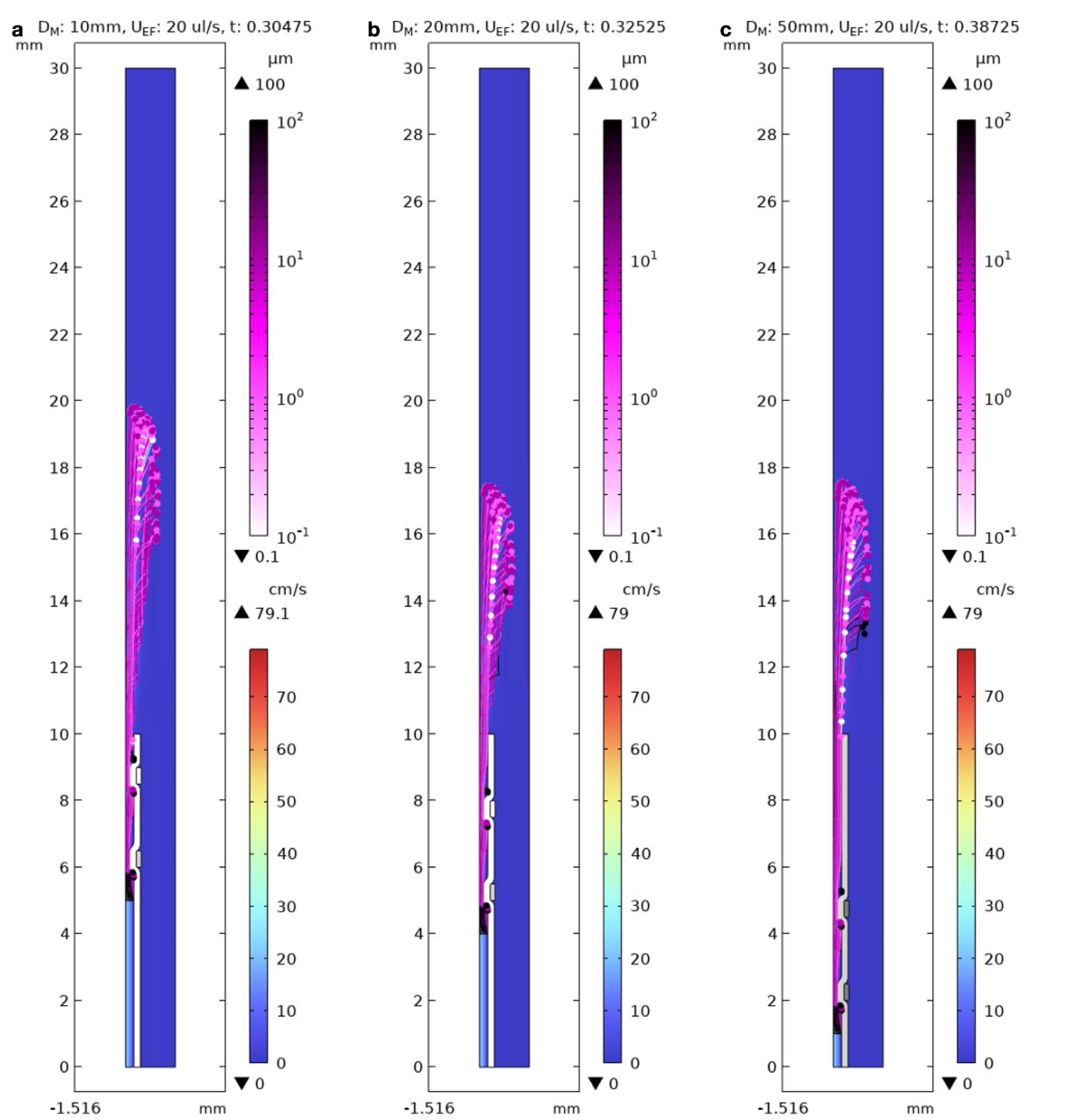


Figure S8. Parametric numerical simulations to optimize the catheter design (i.e., distance from the ring magnet to the catheter tip) for effectively ejecting (at an ejection flow rate of 20 µl s^-1^) the particles from the reservoir of the catheter tip. (a-c) Surface velocity maps and particles trajectories at the time-point when the full-ejection of the reservoir is completed for various designs having a catheter tip to magnet distance of (a) 1 mm, (b) 2 mm, and (c) 5 mm, respectively.


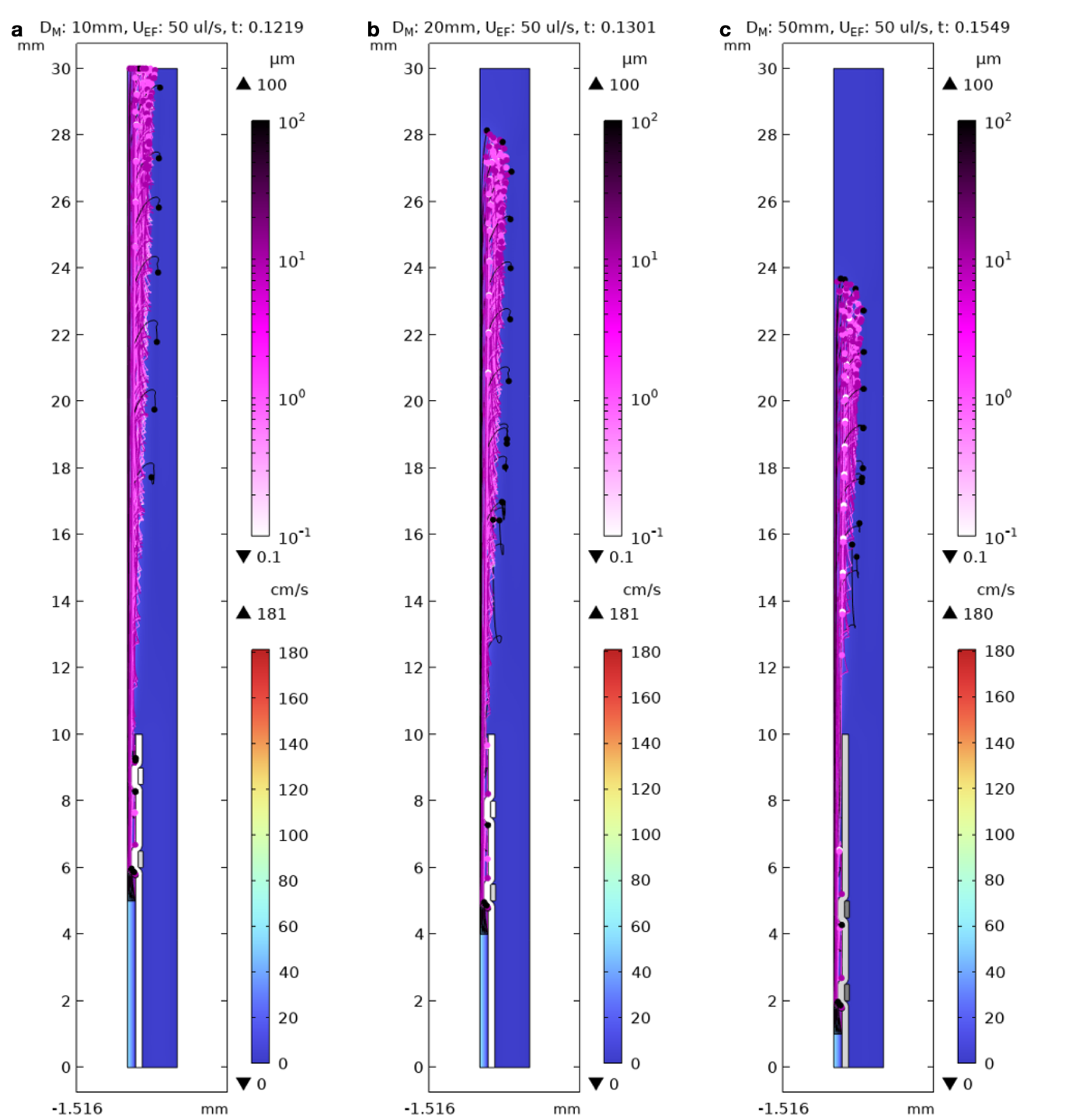


Figure S9. Parametric numerical simulations to optimize the catheter design (i.e., distance from the ring magnet to the catheter tip) for effectively ejecting (at an ejection flow rate of 50 µl s^-1^) the particles from the reservoir of the catheter tip. (a-c) Surface velocity maps and particles trajectories at the time-point when the full-ejection of the reservoir is completed for various designs having a catheter tip to magnet distance of (a) 1 mm, (b) 2 mm, and (c) 5 mm, respectively.


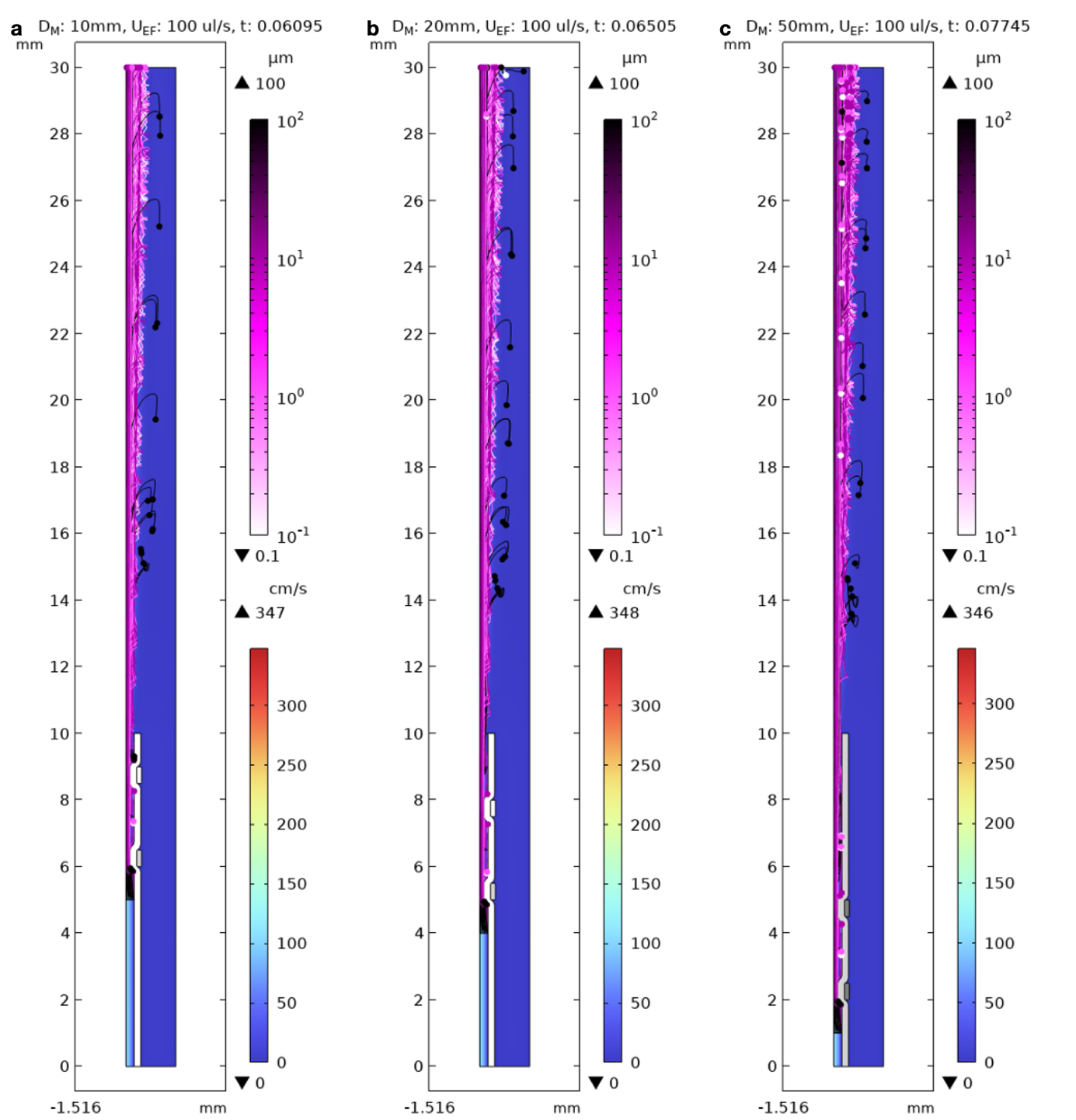


Figure S10. Parametric numerical simulations to optimize the catheter design (i.e., distance from the ring magnet to the catheter tip) for effectively ejecting (at an ejection flow rate of 100 µl s^-1^) the particles from the reservoir of the catheter tip. (a-c) Surface velocity maps and particles trajectories at the time-point when the full-ejection of the reservoir is completed for various designs having a catheter tip to magnet distance of (a) 1 mm, (b) 2 mm, and (c) 5 mm, respectively.


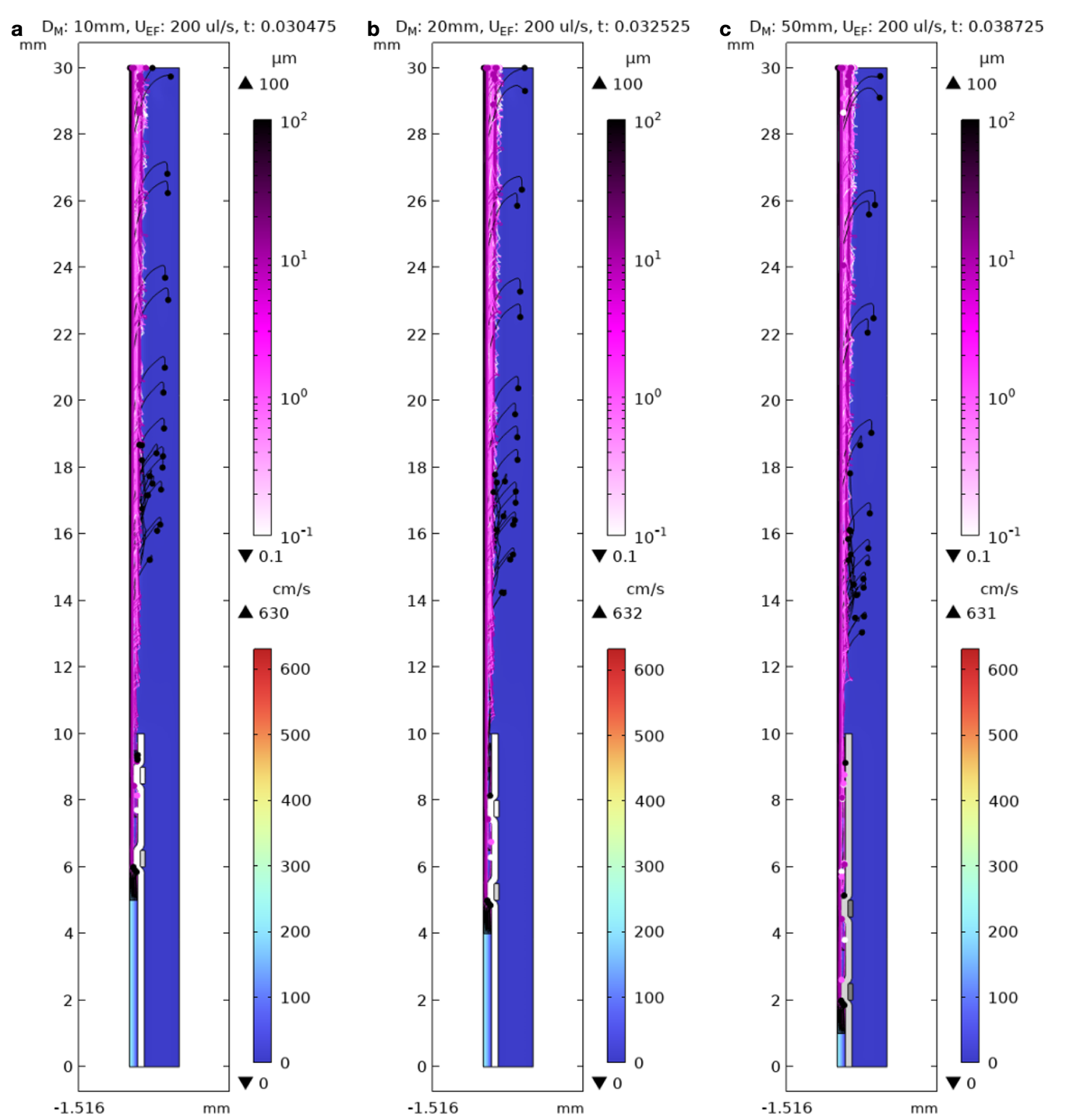


Figure S11. Parametric numerical simulations to optimize the catheter design (i.e., distance from the ring magnet to the catheter tip) for effectively ejecting (at an ejection flow rate of 200 µl s^-1^) the particles from the reservoir of the catheter tip. (a-c) Surface velocity maps and particles trajectories at the time-point when the full-ejection of the reservoir is completed for various designs having a catheter tip to magnet distance of (a) 1 mm, (b) 2 mm, and (c) 5 mm, respectively.


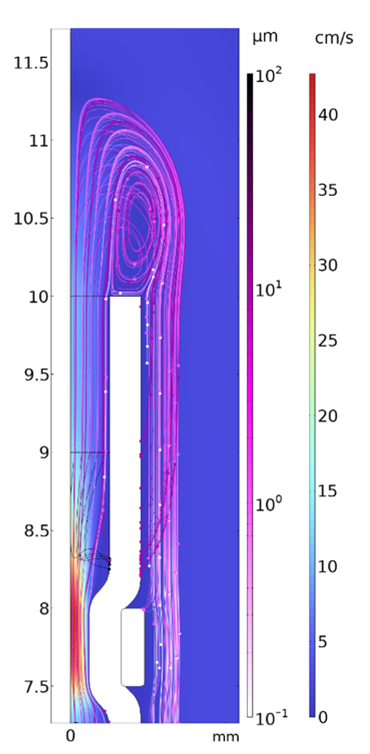


Figure S12. Trajectories of the particle aggregates at t = 0.5 s for an ejection flow of 10 µl s^-1^ and counter-flow of 2.5 cm s^-1^. The low ejection flow rate is ineffective in releasing the IONPs further from the catheter and results in their undesired backflow around the catheter tip.


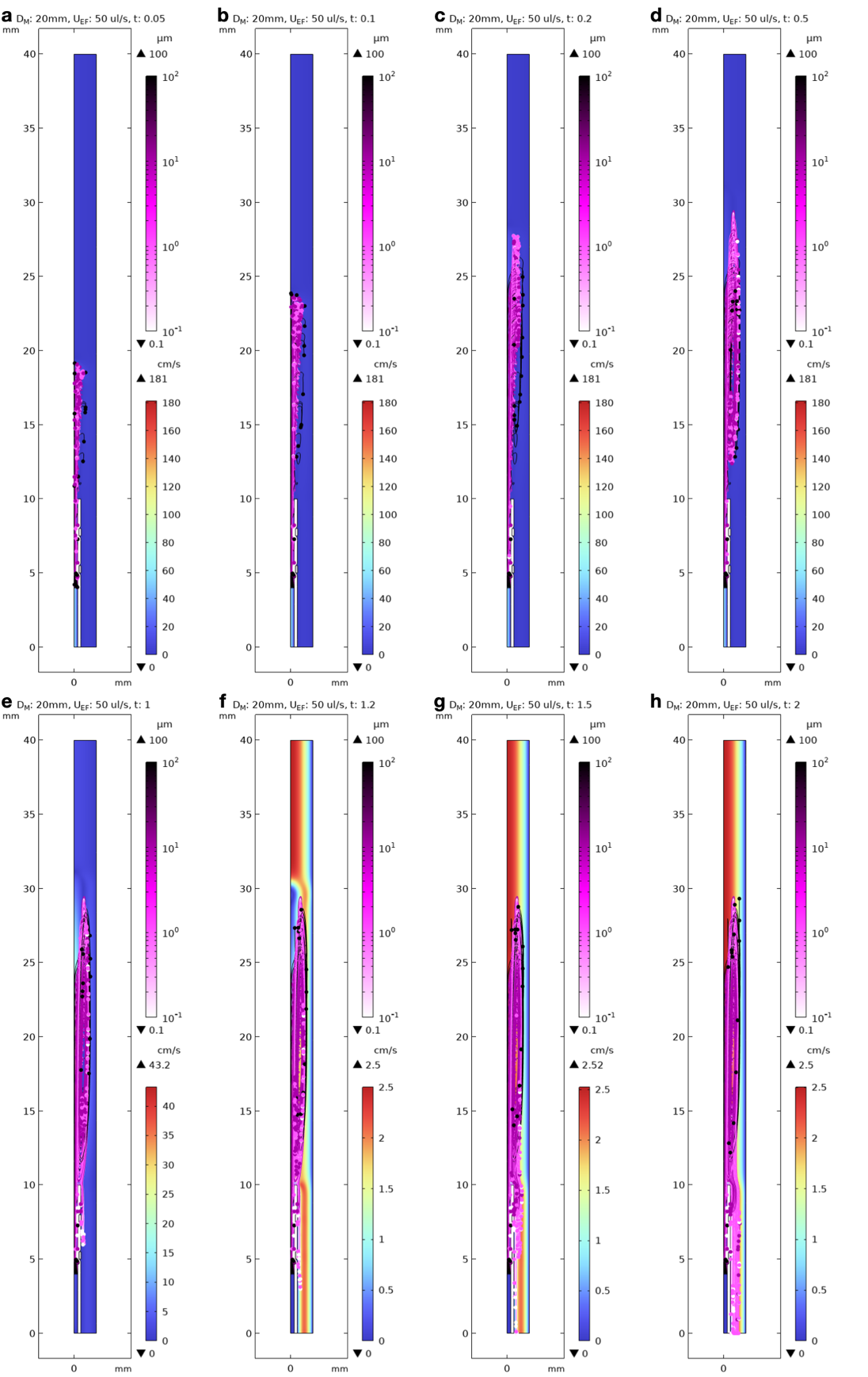


Figure S13. Transient numerical simulations that investigate the trajectories of aggregates ejected at a flow rate of 50 µl s^-1^ against a counter flow (2.5 cm s^-1^) under the effect of axially applied magnetic field gradient, *𝛻B_z_* = 0.3 mT mm^-1^. (a-e) Presenting the velocity maps and aggregates trajectories at different time points (0.05, 0.1, 0.2, 0.5, and 1 s, respectively) during the 1 s ejection event, including ramp-up, ejection at constant flow rate (50 µl s^-1^), and ramp-down phases. (f-g) Presenting the velocity maps and aggregates trajectories at different time points (1.2, 1.5, and 2 s, respectively) after the 1 s ejection event is completed, hence the trajectories of particles are determined by the combined effect of counter flow (2.5 cm s^-1^) and axially applied magnetic field gradient, *𝛻B_z_* = 0.3 mT mm^-1^.


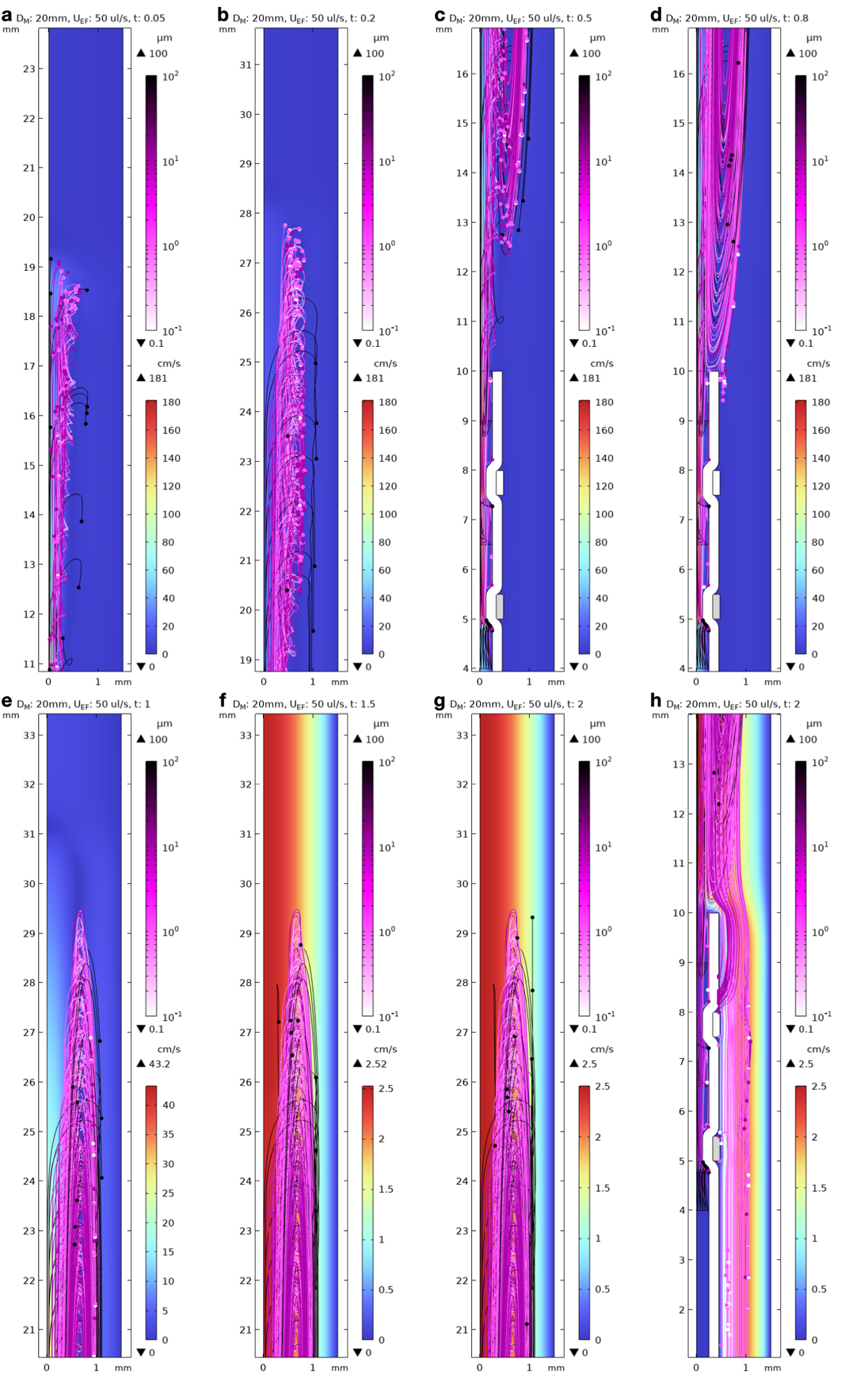


Figure S14. Close look of the aggregates trajectories (in Figure S13) ejected at a flow rate of 50 µl s^-1^ against a counter flow (2.5 cm s^-1^) under the effect of axially applied magnetic field gradient, *𝛻B_z_* = 0.3 mT mm^-1^. (a-e) Presenting the velocity maps and aggregates trajectories at different time points (0.05, 0.2, 0.5, 0.8, and 1 s, respectively) during the 1 s ejection event, including ramp-up, ejection at constant flow rate (50 µl s^-1^) and ramp-down phases. (f-h) Presenting the velocity maps and aggregates trajectories at different time points (1.5 s and 2 s) after the 1 s ejection event is completed, hence the trajectories of aggregates are determined by the combined effect of counter flow (2.5 cm s^-1^) and axially applied magnetic field gradient, *𝛻B_z_* = 0.3 mT mm^-1^. Specifically, focusing on the aggregates (f-g) away from the catheter and (h) around the catheter.


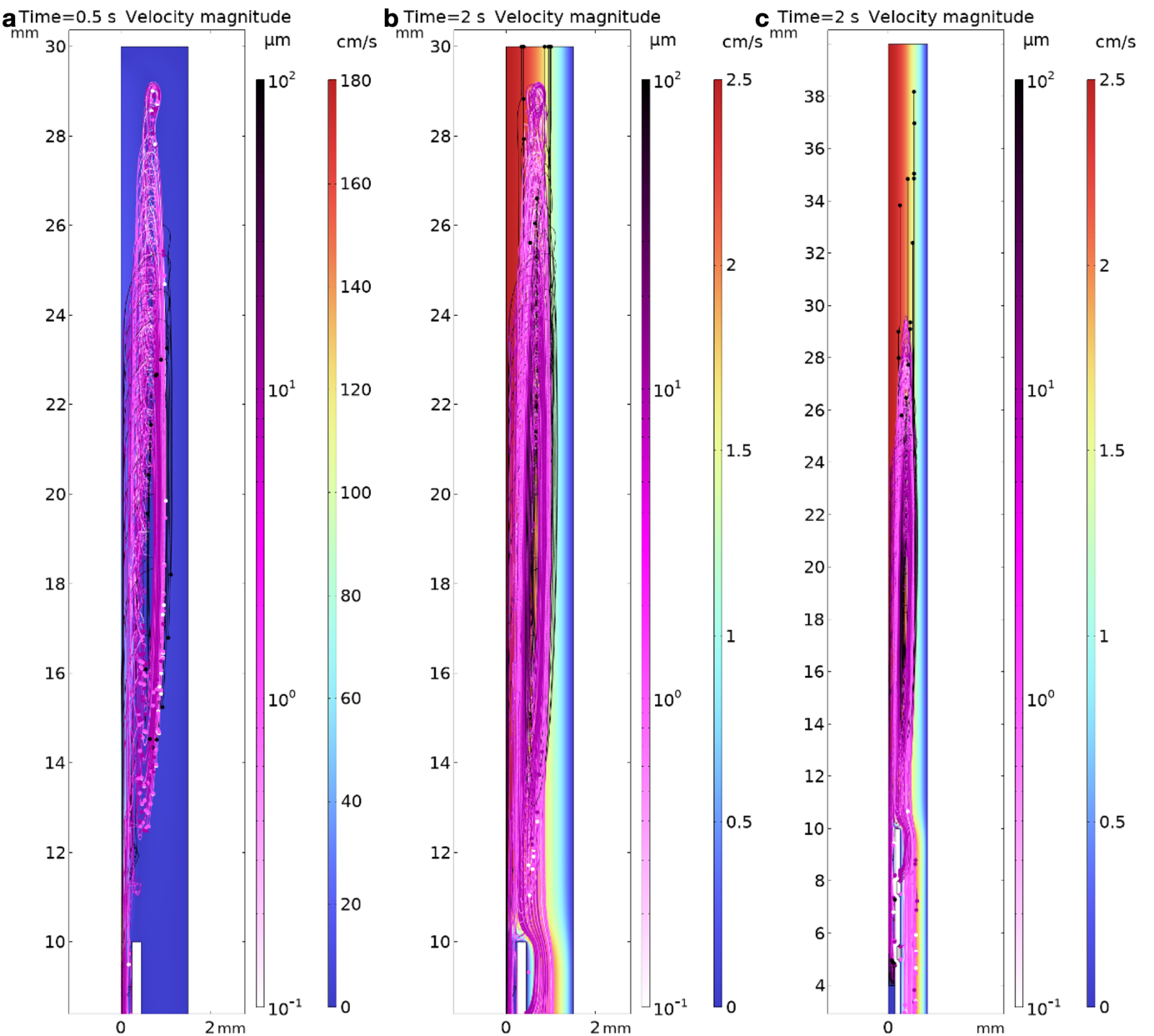


Figure S15. Transient numerical simulations that investigate the trajectories of aggregates ejected at a flow rate of 50 µl s^-1^ against a counter flow (2.5 cm s^-1^) under the effect of axially applied magnetic field gradient, *𝛻B_z_* = 0.5 mT mm^-1^. (a) Presenting the velocity maps and aggregates trajectories at t = 0.5 s during the ejection (50 µl s^-1^). (b-c) Presenting the velocity maps and aggregates trajectories at t = 2 s, after the 1 s ejection event is completed, hence the trajectories of aggregates are determined by the combined effect of counter flow (2.5 cm s^-1^) and axially applied magnetic field gradient, *𝛻B_z_* = 0.5 mT mm^-1^. Note that to better investigate the magnetic motion of aggregates, the size of simulation domain is increased from (b) 30 mm to (c) 40 mm, allowing to clearly observe the magnetic navigation of IONP aggregates (≥100 µm) against a counter flow of 2.5 cm s^-1^.


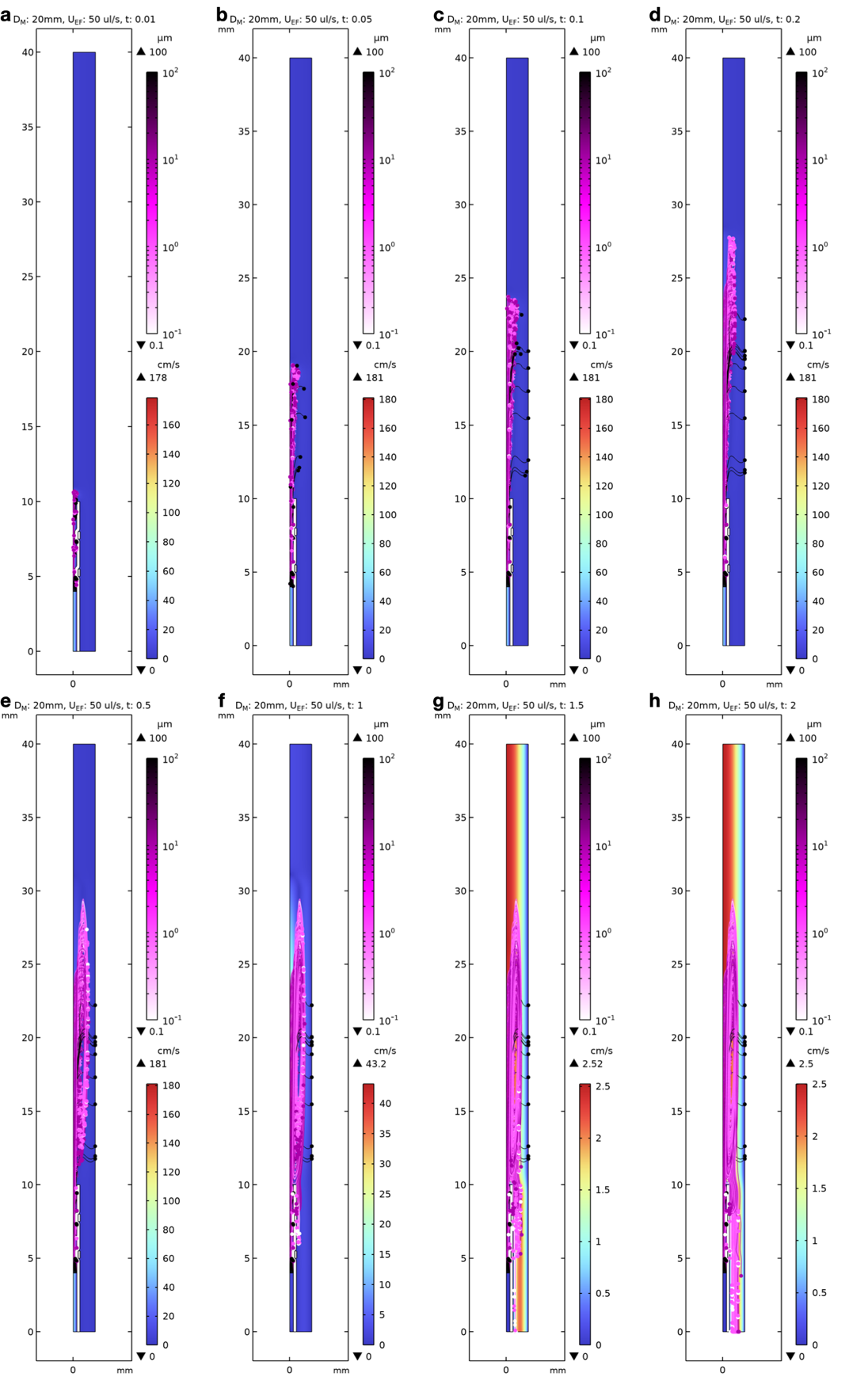


Figure S16. Transient numerical simulations that investigate the trajectories of aggregates ejected at a flow rate of 50 µl s^-1^ against a counter flow (2.5 cm s^-1^) under the combined effect of radially and axially applied magnetic field gradients, respectively *𝛻B_r_* = 0.3 mT mm^-1^ and *𝛻B_z_* = 0.5 mT mm^-1^. (a-f) Presenting the velocity maps and aggregates trajectories at different time points (0.01, 0.05, 0.1, 0.2, 0.5, and 1 s, respectively) during the 1 s ejection event, including ramp-up, ejection at constant flow rate (50 µl s^-1^), and ramp-down phases. (g-h) Presenting the velocity maps and aggregates trajectories at different time points (1.5 s and 2 s) after the 1 s ejection event is completed, hence the trajectories of aggregates are determined by the combined effect of counter flow (2.5 cm s^-1^) and applied magnetic field gradients, *𝛻B_r_* = 0.3 mT mm^-1^ and *𝛻B_z_* = 0.5 mT mm^-1^. Note that due to the *stick* boundary condition (which is utilized to decrease the computational cost) on the domain walls the aggregates that touch to the domain boundary stay at the location. Nevertheless, their curved trajectories and results presented in Figure S13-S15 suggest that these aggregates still could be navigated with the magnetic field gradients.


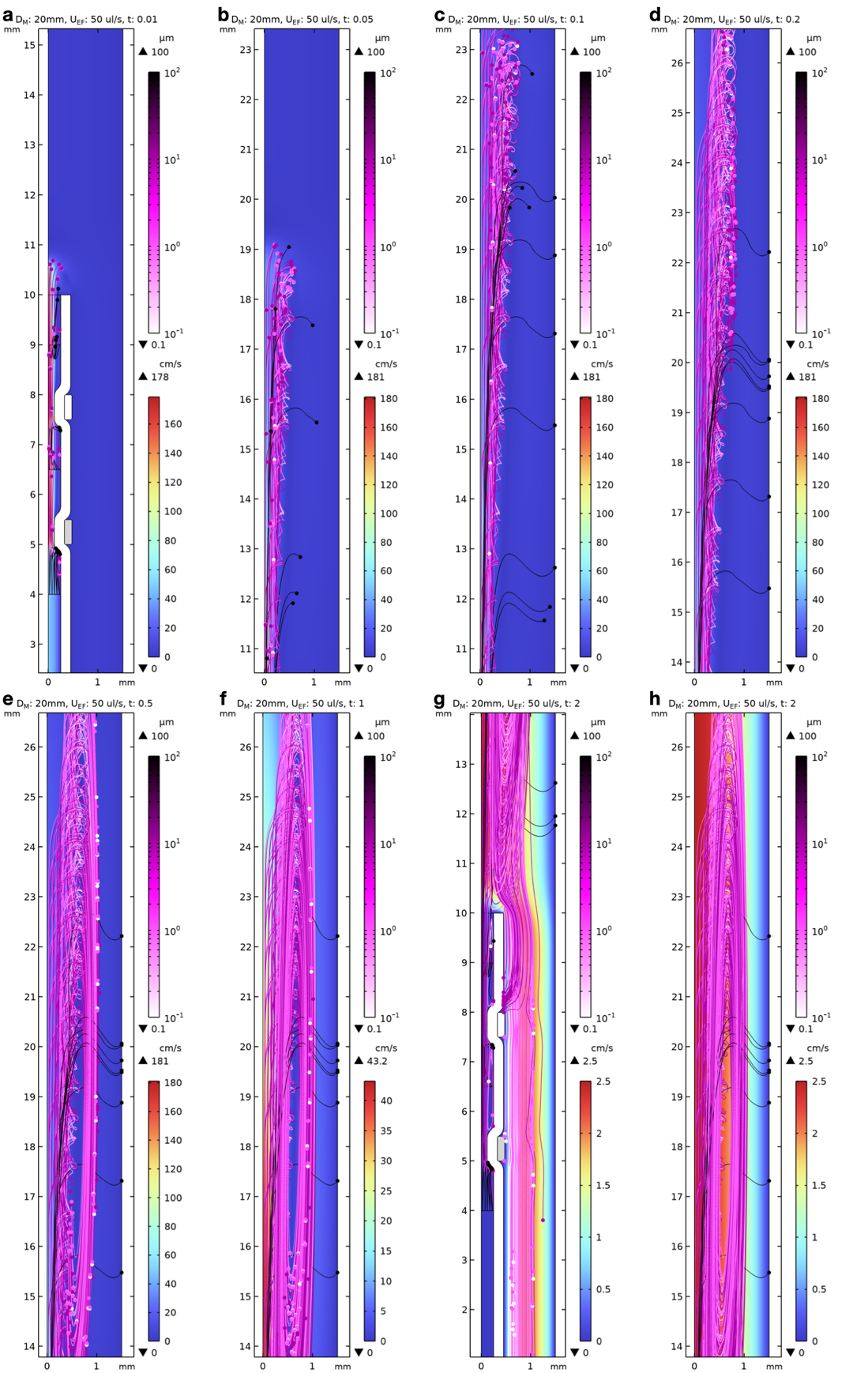


Figure S17. Close look of the aggregates trajectories (in Figure S16) ejected at a flow rate of 50 µl s^-1^ against a counter flow (2.5 cm s^-1^) under the combined effect of radially and axially applied magnetic field gradients, respectively *𝛻B_r_* = 0.3 mT mm^-1^ and *𝛻B_z_* = 0.5 mT mm^-1^. (a-f) Presenting the velocity maps and aggregates trajectories at different time points (0.01, 0.05, 0.1, 0.2, 0.5, and 1 s, respectively) during the 1 s ejection event, including ramp-up, ejection at constant flow rate (50 µl s^-1^) and ramp-down phases. (g-h) Presenting the velocity maps and aggregates trajectories at t = 2 s (g) around the catheter and (h) away from catheters tip, after the 1 s ejection event is completed, hence the trajectories of aggregates are determined by the combined effect of counter flow (2.5 cm s^-1^) and applied magnetic field gradients, *𝛻B_r_* = 0.3 mT mm^-1^ and *𝛻B_z_* = 0.5 mT mm^-1^. Note that due to the *stick* boundary condition (which is utilized to decrease the computational cost) on the domain walls the aggregates that touch to the domain boundary stays at the location. Nevertheless, their curved trajectories and results presented in Figure S13-S15 suggest that these aggregates still could be navigated with the magnetic field gradients.


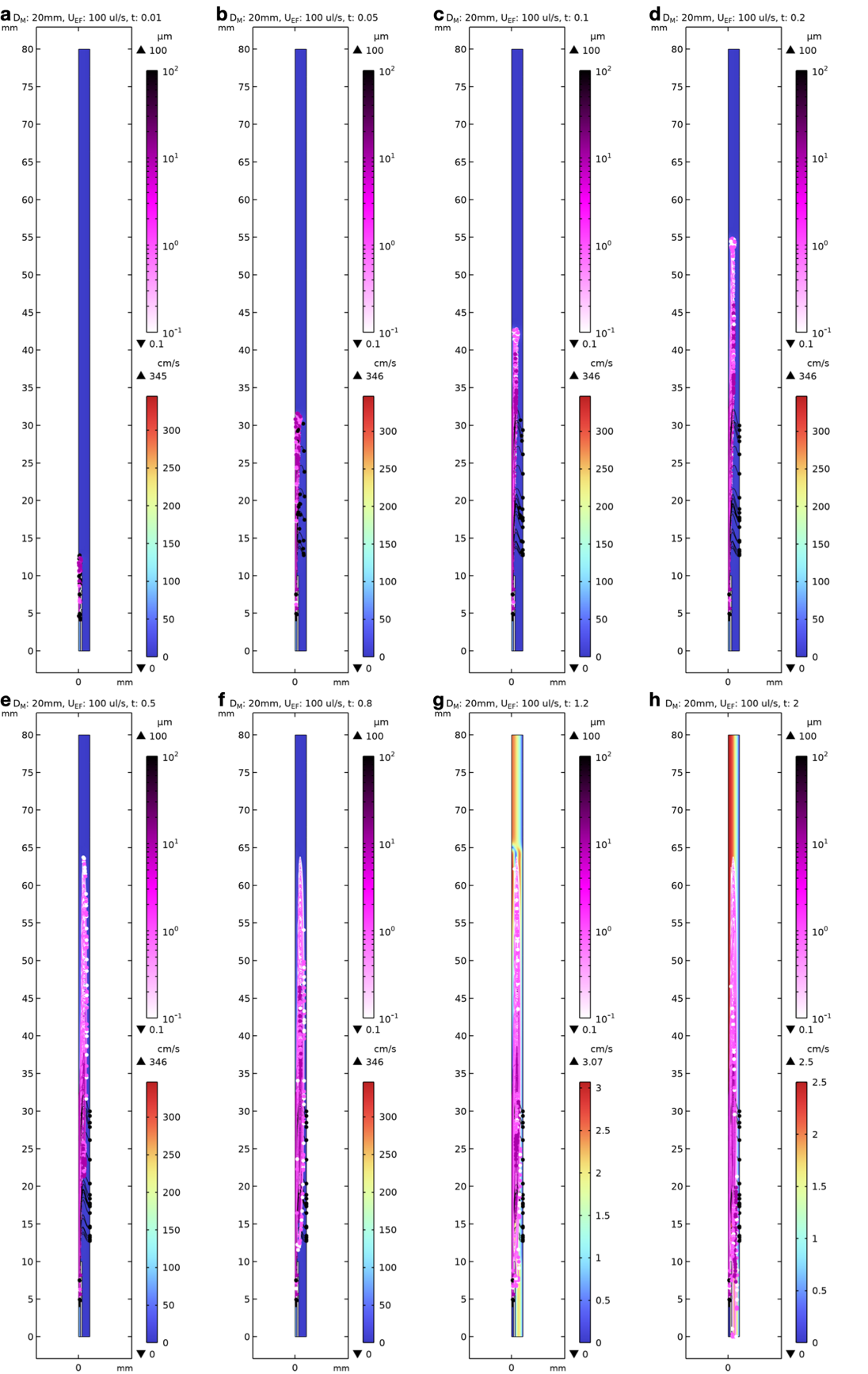


Figure S18. Transient numerical simulations that investigate the trajectories of aggregates ejected at a flow rate of 100 µl s^-1^ against a counter flow (2.5 cm s^-1^) under the combined effect of radially and axially applied magnetic field gradients, respectively *𝛻B_r_* = 0.3 mT mm^-1^ and *𝛻B_z_* = 0.5 mT mm^-1^. (a-f) Presenting the velocity maps and aggregates trajectories at different time points (0.01, 0.05, 0.1, 0.2, 0.5, and 0.8 s, respectively) during the 1 s ejection event, including ramp-up, ejection at constant flow rate (100 µl s^-1^) and ramp-down phases. (g-h) Presenting the velocity maps and aggregates trajectories at different time points (1.2 s and 2 s) after the 1 s ejection event is completed, hence the trajectories of aggregates are determined by the combined effect of counter flow (2.5 cm s^-1^) and applied magnetic field gradients, *𝛻B_r_* = 0.3 mT mm^-1^ and *𝛻B_z_* = 0.5 mT mm^-1^. Note that due to the *stick* boundary condition (which is utilized to decrease the computational cost) on the domain walls the aggregates that touch to the domain boundary stays at the location. Nevertheless, their curved trajectories and results presented in Figure S13-S15 suggest that these aggregates still could be navigated with the magnetic field gradients.


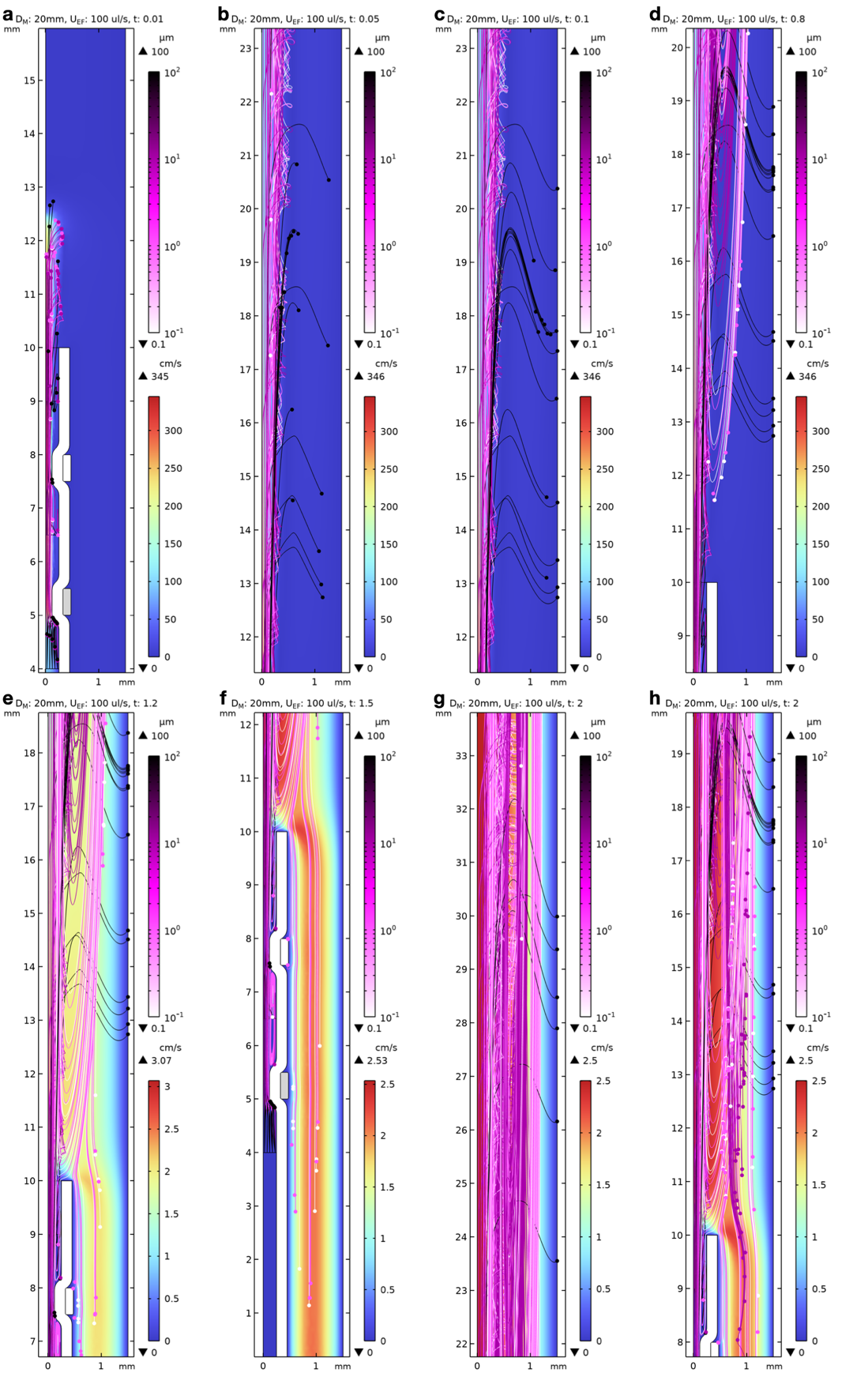


Figure S19. Close look of the aggregates trajectories (in Figure S18) ejected at a flow rate of 100 µl s^-1^ against a counter flow (2.5 cm s^-1^) under the combined effect of radially and axially applied magnetic field gradients, respectively *𝛻B_r_* = 0.3 mT mm^-1^ and *𝛻B_z_* = 0.5 mT mm^-1^. (a-d) Presenting the velocity maps and aggregates trajectories at different time points (0.01, 0.05, 0.1, and 0.8 s, respectively) during the 1 s ejection event. (e-h) Presenting the velocity maps and aggregates trajectories at different time points (1.2, 1.5, and 2 s) after the ejection event is completed, focusing on the region (e, f, h) around the catheter tip and (g) away from the catheters tip. Note that due to the *stick* boundary condition (which is utilized to decrease the computational cost) on the domain walls the aggregates that touch to the domain boundary stays at the location. Nevertheless, their curved trajectories and results presented in Figure S13-S15 suggest that these aggregates still could be navigated with the magnetic field gradients. Moreover, increased ejection flow rate (100 µl s^-1^) results in ejection of more aggregates to further away locations.


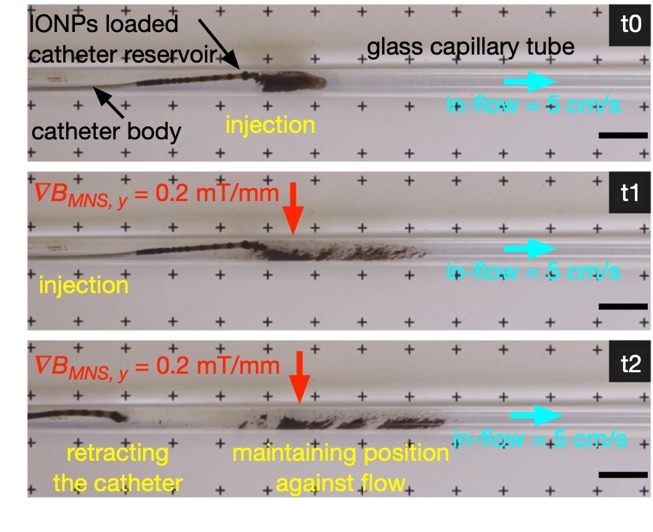


Figure S20. Experimental results of in-flow ejection of IONPs inside the capillary tube under the effect of magnetic control. External flow in the same direction as the ejection is 5 cm s^-1^. By applying a magnetic field gradient (*𝛻B_MNS,y_* = 0.2 mT mm^-1^) that is distributed along the whole microchannel, IONPs maintain the ejected position against the external flow (Scale bar: 10 mm).


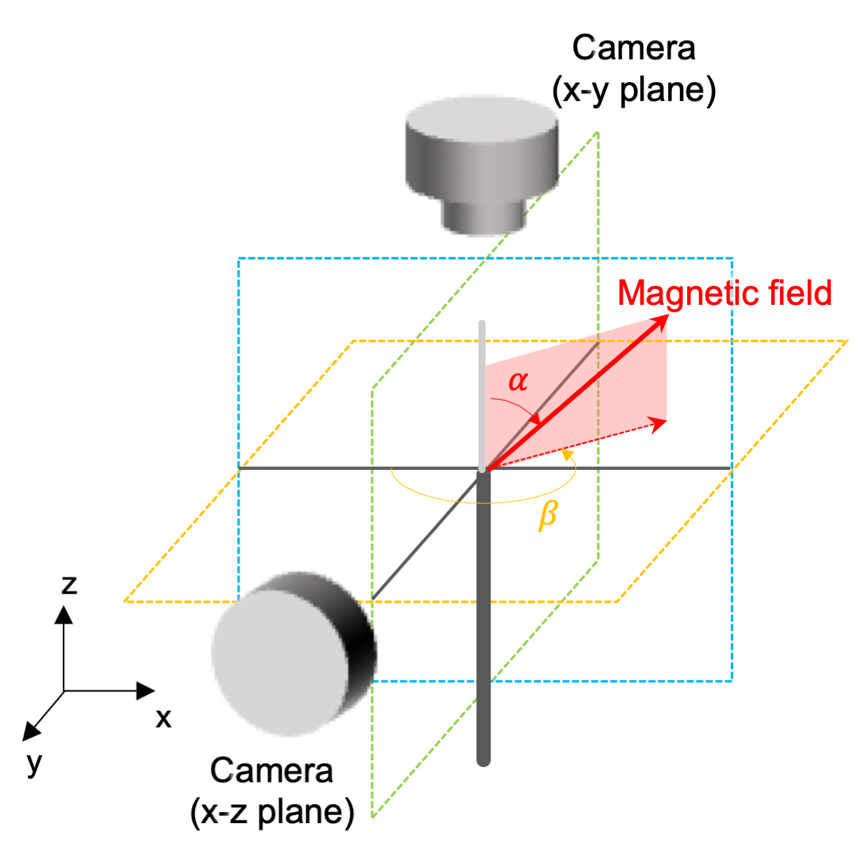


Figure S21. Experimental configuration for evaluation of the magnetic microcatheter steerability. Note that the coordinate and origin of the magnetic field angles 𝛼 and 𝛽 are defined for the clear visualization. The actual magnetic field inputs in the eMNS are AA = 90º – 𝛼 and IA = 𝛽 – 90º (in Supporting Video 1-4).


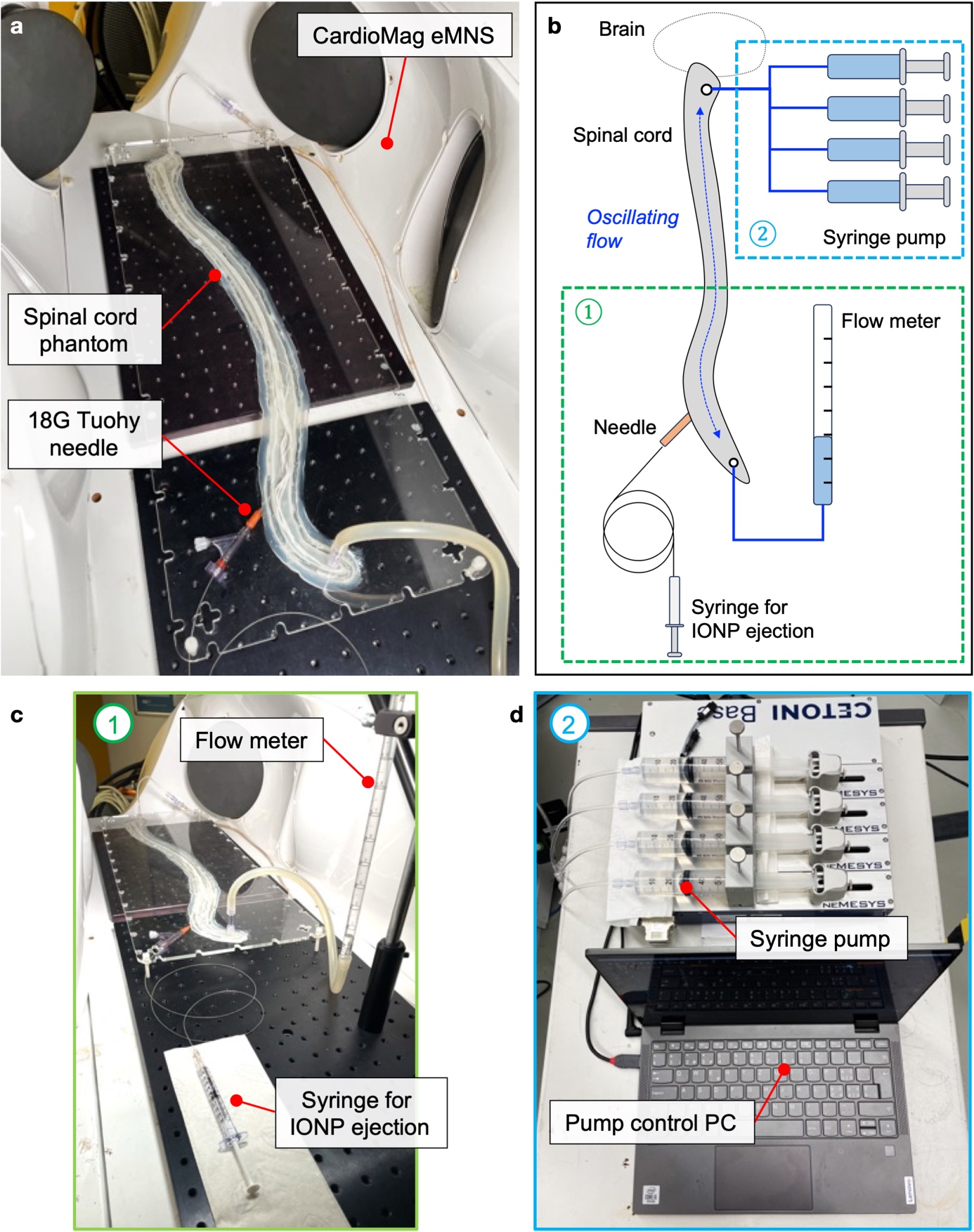


Figure S22. *In-vitro* proximity injection experiments within the 3-D printed spinal cord phantom that mimics the spinal subarachnoid space and oscillating flow of cerebrospinal fluid (CSF). (a) Picture of the spinal cord phantom placed on CardioMag eMNS. (b) Schematic representation of experimental setup. (c-d) Detailed pictures of the experimental setup showing (c) phantom model of spinal cord placed on CardioMag eMNS, syringe to pump water for ejecting the IONPs from the reservoir of microcatheter, flowmeter to visualize the mimicked oscillating CSF flow; as well as (d) syringe pumps used to realize the oscillating DI water flow (2.5 cm s^-1^ at 1 Hz) inside the phantom model.

**Supporting Tables**

Table S1. Parameter set for numerical simulations without counter-flow; resulting 15 independent simulations (presented in Figure S7-S11) to optimize the catheter tip design (magnet to tip distance) and ejection flow rates.

| **Parameter** | **Unit** | **Values** |
| --- | --- | --- |
| Particle diameter  (uniform distribution for of size) | µm | 0.1, 1, 10, 100 |
| CSF flow velocity  (counter flow) | cm s^-1^ | 0 |
| Ejection flow rate | µl s^-1^ | 10, 20, 50, 100, 200 |
| eMNS, Magnetic field strength | mT | 30 |
| eMNS, Magnetic field gradient | mT mm**^-1^** | 0 |
| Magnet to tip distance | mm | 1, 2, 5 |

Table S2. Results of parametric numerical simulations (presented in Figure S7-S11) showing the ejected particle percentages based on ejection flow rates (ranging from 10 µl s^-1^ to 200 µl s^-1^) for three different catheter designs (i.e., magnet to tip distance is 1 mm, 2 mm, and 3 mm, respectively for short, medium, and long tip designs). Number of total particles simulated in each simulation is 260.

| **Ejection**  **flow rate**  **(µl s^-1^)** | **Percentage of ejected particles**  **using short tip**  **(magnet to tip distance = 1 mm)** | **Percentage of ejected particles**  **using medium tip**  **(magnet to tip distance = 2 mm)** | **Percentage of ejected particles**  **using long tip**  **(magnet to tip distance = 5 mm)** |
| --- | --- | --- | --- |
| 10 | 61.2% | 61.5% | 62.7% |
| 20 | 65.0% | 66.5% | 67.7% |
| 50 | 72.3% | 73.8% | 75.4% |
| 100 | 79.2% | 81.2% | 80.4% |
| 200 | 84.6% | 86.5% | 81.9% |

Table S3. Parameter set for numerical simulations under the combined effect of counter-flow (2.5 cm s^-1^); and magnetic field gradients; resulting 5 independent simulations (presented in Figure S12-S19) with different parameter combinations.

| **Parameter** | **Unit** | **Values** |
| --- | --- | --- |
| Particle diameter  (uniform distribution for of size) | µm | 0.1, 1, 10, 100 |
| CSF flow velocity  (counter flow) | cm s^-1^ | 2.5 |
| Ejection flow rate | µl s^-1^ | 10, 50, 100 |
| eMNS, Magnetic field strength | mT | 30 |
| eMNS, Magnetic field gradient | mT mm**^-1^** | 0.3 (𝛻*B_z_*), 0.5 (𝛻*B_z_*), combination of 0.5 (𝛻*B_z_*) and 0.3 (𝛻*B_r_*) |
| Magnet to tip distance | mm | 2 |

Supporting References

[1] F. H. L. Starsich, I. K. Herrmann, S. E. Pratsinis, *Annu. Rev. Chem. Biomol. Eng.* **2019**, *10*, 155.

[2] M. Mahmoudi, S. Sant, B. Wang, S. Laurent, T. Sen, *Advanced Drug Delivery Reviews* **2011**, *63*, 24.

[3] M. Mahmoudi, M. A. Sahraian, M. A. Shokrgozar, S. Laurent, *ACS Chem. Neurosci.* **2011**, *2*, 118.

[4] S. Shanehsazzadeh, A. Lahooti, M. J. Hajipour, M. Ghavami, M. Azhdarzadeh, *Colloids and Surfaces B: Biointerfaces* **2015**, *136*, 1107.

[5] H. McConnell, J. Bianchine, A. Wilder-Smith, in *Cerebrospinal Fluid in Neurology and Psychiatry* (Eds.: H. McConnell, J. Bianchine), Springer US, Boston, MA, **1994**, pp. 57–109.

[6] E. Lueshen, I. Venugopal, J. Kanikunnel, T. Soni, A. Alaraj, A. Linninger, *Nanomedicine* **2014**, *9*, 1155.

[7] L. R. Sass, M. Khani, G. C. Natividad, R. S. Tubbs, O. Baledent, B. A. Martin, *Fluids Barriers CNS* **2017**, *14*, 36.
